# Immune imbalance and autoimmune mechanisms in plasma cell mastitis: current perspectives

**DOI:** 10.3389/fcell.2025.1727675

**Published:** 2026-01-16

**Authors:** Bingjie Xue, Yuang Jiang, Yan Lei, Xin Liu, Jing Li, Yunxiang Li, Binyue Zhang, Yanhong Wang, Hongyan Jia

**Affiliations:** 1 Department of First Clinical Medicine, Shanxi Medical University, Taiyuan, Shanxi, China; 2 Department of Breast Surgery, First Hospital of Shanxi Medical University, Taiyuan, Shanxi, China; 3 Department of Microbiology and Immunology, School of Basic Medical Sciences, Shanxi Medical University, Taiyuan, Shanxi, China; 4 Key Laboratory of Cellular Physiology (Shanxi Medical University), Ministry of Education, Taiyuan, Shanxi, China

**Keywords:** adaptive immunity, complement system, exosome, immune imbalance, innate immunity, plasma cell mastitis (PCM), review

## Abstract

Plasma cell mastitis (PCM), also termed ductal dilatation or periductal mastitis (PDM), is a benign, non-bacterial inflammatory breast disorder predominantly affecting non-lactating women. Its incidence continues to rise, yet diagnosis and therapeutic approaches remain under development. Consequently, elucidating the pathogenesis is essential for identifying the disease’s root cause and facilitating breakthroughs. PCM is increasingly regarded as an autoimmune disorder, highlighting the crucial role of immune factors in its development; however, the precise immune mechanisms involved remain incompletely understood. Against these backgrounds, this review aims to: (1) discuss the contributions of various innate and adaptive immune cells within the PCM-associated immune microenvironment; (2) describe the relationship between imbalances in key immune cells (including M1/M2 macrophages, Th1/Th2 lymphocytes, Th17/Treg cells) and the progression of PCM; (3) explore potential future research areas, the underlying immunopathogenesis of PCM, and prospective therapeutic targets, the activation of the complement system, regulation of immune homeostasis, the role of immune cell circadian rhythms, and modulation of key immune pathways.

## 1 Introduction

Plasma cell mastitis (PCM), also termed ductal dilatation or periductal mastitis (PDM), is a chronic non-bacterial inflammatory breast disorder (Xu et al., 2023; Gopalakrishnan Nair et al., 2015; Liu et al., 2017). It is frequently classified alongside granulomatous mastitis (GLM) within the spectrum of non-lactating mastitis (NPM) (Zhang et al., 2022). PCM primarily affects young and middle-aged women, with occasional cases in men (Long et al., 2021; Ashworth et al., 1985). Although accounting for only 4%–5% of benign breast diseases, its incidence has risen in recent years, with a trend toward younger patients. Clinical manifestations include breast erythema, induration, pain, abscess formation, and periareolar duct fistulae (Zhang et al., 2018). Histopathologically, the characteristic features include dense plasma cell infiltration, dilation of peripheral mammary ducts, and damage to mammary epithelial cells (Zhang et al., 2022; Wang et al., 2019; Li X. Q. et al., 2022). The disease frequently follows a protracted clinical course with high recurrence rates (Ren et al., 2021), posing significant clinical challenges in non-lactating women.

Diagnosis primarily relies on core needle biopsy. However, overlapping clinical and radiological features with breast malignancies (Ortiz-Mendoza et al., 2018; Chen et al., 2020; Zhu et al., 2019; Yang et al., 2018) and GLM (Kong et al., 2020) contribute to frequent misdiagnosis. Some studies have shown that multimodal approaches could improve the diagnostic accuracy of PCM. They include combining evaluation of routine breast screening ultrasound and mammography (Hu and Huang, 2020), combining assessment of ultrasonography and haematological analysis (Zheng Y. et al., 2022), advanced ultrasound multimodal imaging methods (Li C. et al., 2022), and AI-assisted technology (Zhou et al., 2022), *etc.* However, PCM does not exhibit specific clinical or imaging characteristics, and it manifests with diverse symptoms and considerable individual variability; therefore, misdiagnosis remains prevalent. Therapeutic management encounters comparable challenges. Presently, surgical excision constitutes the primary modality of treatment (Zhang et al., 2018); however, the surgery not only results in a change in breast morphology but also constrains the ability to control recurrence rates (Zhou et al., 2023). For patients with early PCM who do not have a bacterial infection, antibiotic treatment is generally ineffective. Though hormone therapy can be used in PCM (Faccin et al., 2016), it tends to recur after stopping the medication (Zhou et al., 2023). Moreover, it is challenging to attain a cure through medication for patients suffering from breast conditions indentation (Xu et al., 2023). Furthermore, anti-tuberculosis medications must be administered with caution due to their potential side effects, such as nephrotoxicity (Zhou et al., 2024a). Chinese medical treatment can mitigate risks, including alterations in breast shape, impacts on breastfeeding function, increased resection area, and challenges in recurrence reduction surgery (Wu et al., 2022). Nonetheless, it exhibits a slower onset of action and necessitates a higher level of patient compliance. Furthermore, for PCM patients with established abscesses or sinus tracts, its efficacy remains markedly limited. Presently, several innovative therapeutic options are still in the exploratory phase stage (Zhou et al., 2023).

Given the many challenges in diagnosing and treating PCM, it is particularly important to explore its pathogenesis in depth to develop more effective treatments targeting the root cause. Present studies show that obesity, smoking, and trauma may be risk factors for the development of PCM. At the same time, these conclusions are mainly based on case reports and an adequate epidemiological basis is lacking (Gollapalli et al., 2010; Risager and Bentzon, 2010). Emerging research increasingly supports an immune-mediated etiology (Xie et al., 2025; Zhou et al., 2025), with dysregulated immune responses playing a central role (Faccin et al., 2016; Liu et al., 2015; Clark et al., 2015). This review synthesizes evidence accumulated over the past decade regarding immune mechanisms in PCM. This review categorizes the involved immune mechanisms into three levels of evidence: (1) Direct evidence: cells, molecules, or pathways that demonstrate significant alterations in tissues or body fluids from PCM patients; (2) Indirect/associated evidence: findings derived from broader NPM or idiopathic granulomatous mastitis (IGM), which may be applicable to PCM; and (3) Hypothetical/inferred evidence: mechanisms proposed based on other autoimmune diseases or models of sterile inflammation that have not yet been validated in PCM. This framework aims to clearly distinguish between established findings and areas that require further investigation.

## 2 The characteristic of the immune microenvironment in PCM

### 2.1 Pathological characteristics of PCM and the “sterile inflammation” hypothesis

Normal breast tissue comprises independently established leaflet structures. The interlobular areas are delineated by connective tissue; their ducts do not exhibit abnormal dilatation; the microvilli of the epithelial cells are systematically organized. Cellular morphology remains normal, and intercellular spaces are regular. In tissues affected by PCM lesions, the basement membrane of breast ductal epithelial cells was disrupted, resulting in reduced cellular space and bridging granule structures. The ducts exhibited significant dilation, and a dense infiltration of immune cells was observed within them (Zhang et al., 2018). Concurrently, there was a notable upregulation of inflammatory factor expression (Liu et al., 2017). These pathological characteristics strongly indicate that an abnormal and heightened immune-inflammatory response constitutes the central pathological mechanism in PCM.

PCM is frequently characterized as a form of “sterile inflammation”, and its manifestations are closely associated with the migration and recruitment of leukocytes. Unlike pathogen-induced stimulation, which activates immune responses through pathogen-associated molecular patterns (PAMPs) mediated by pattern recognition receptors (PRRs), sterile inflammation is primarily driven by damage-associated molecular patterns (DAMPs) released from injurious factors (Zindel and Kubes, 2020). DAMPs are intracellular components that are released into the extracellular space during tissue injury, necrosis, or cellular stress. They can be recognized and activated by PRRs—for example, Toll-like receptors (TLRs) expressed on the surface of innate immune cells—as well as by non-PRR receptors. Thereby triggering and participating in both innate and adaptive immune inflammatory responses (Dwyer and Turnquist, 2021). In PCM, it is hypothesized that initial damage to the mammary duct epithelium—induced by factors such as trauma, ductal obstruction, hyperprolactinemia, or congenital nipple inversion—may represent the major source of DAMP release. This, in turn, activates an abnormal immune response targeting self-tissues, thereby supporting the hypothesis that PCM is an autoimmune disease (Faccin et al., 2016; Xie et al., 2025; Peters and Schuth, 1989).

### 2.2 Overall immune response in PCM: cascade activation of innate and adaptive immunity

A substantial body of mechanistic research has demonstrated that dysregulation of both innate and adaptive immune responses plays a pivotal role in the pathogenesis of PCM.

As the primary immunological barrier, innate immune cells—including macrophages, neutrophils, monocytes, and dendritic cells (DCs)—constitute the first line of defense. Following tissue injury and other insults that induce the release of DAMPs, macrophages and DCs within the mammary tissue rapidly sense and recognize these signals. Macrophages then differentiate into classically activated macrophages (M1 macrophages), which exert pro-inflammatory effects by secreting large quantities of pro-inflammatory mediators and recruiting neutrophils and monocytes to migrate to the lesion site (Zhou et al., 2024b; Shapouri-Moghaddam et al., 2018). In addition, during the later stages of inflammation, these cells further differentiate into alternatively activated macrophages (M2 macrophages), thereby promoting the resolution of inflammation and facilitating tissue repair (Yunna et al., 2020). This balance may be disrupted in PCM. As one of the principal antigen-presenting cells (APCs), DCs can associate with major histocompatibility complexes and subsequently present antigens to activate T lymphocytes, thereby initiating adaptive immune responses (Zindel and Kubes, 2020; Liu J. et al., 2023). In parallel, the complement system is also activated in this process. Its activation products (such as C3a and C5a) function as potent chemotactic factors that amplify the recruitment of inflammatory cells. At the same time, the terminal membrane attack complex (MAC) may directly injure ductal epithelial cells (Zhang et al., 2022; Li X. Q. et al., 2022). Neutrophils contribute to the inflammatory response through degranulation, phagocytosis, and the release of neutrophil extracellular traps (NETs) (Zhu et al., 2023; Zhang H. et al., 2024; Zhang et al., 2023; Mihlan et al., 2024; Kim et al., 2023; Alves-Filho et al., 2010). Natural killer (NK) cells secrete interferon-γ (IFN-γ) and tumor necrosis factor (TNF) and can restrain excessive recruitment of other immune cells (Zitti and Bryceson, 2018). In addition, they can recognize specific inflammatory cells, thereby sustaining the activity of nonspecific cellular effectors (Cerwenka and Lanier, 2016).

Adaptive immunity is subsequently activated, with various APCs stimulating T and B lymphocytes through specific antigen presentation. Naïve T lymphocytes are activated and differentiate into distinct subsets, including helper T lymphocytes (Th1, Th2, Th17, and Tfh cells), regulatory T cells (Treg), and cytotoxic T lymphocytes (CTLs) (Dong, 2021; Reina-Campos et al., 2021). These T-cell subsets further shape the inflammatory microenvironment and amplify or modulate immune responses by secreting characteristic cytokine profiles (for example, Th1 cells secrete IFN-γ). Among them, Th1 and Th17 cells are generally associated with tissue destruction and chronic inflammatory responses, whereas Th2 and Treg cells typically exert anti-inflammatory and tissue-repair functions (Nakayama et al., 2017; Sun et al., 2024a; McIntire et al., 1964; Langrish et al., 2005). In PCM, this balance is disrupted, with pro-inflammatory responses becoming predominant (Zhao et al., 2023). In addition, Th2 cells can secrete interleukin-4 (IL-4) and interleukin-13 (IL-13), thereby promoting B lymphocyte proliferation (Howard et al., 1982). Tfh cells, in turn, facilitate B-cell maturation and antibody class switching (Shekhar and Yang, 2012). It is precisely these abnormally proliferating and highly activated plasma cells that produce substantial quantities of immunoglobulins. This represents the most prominent pathological hallmark of PCM—dense plasma cell infiltration in and around the ducts. These plasma cells may further aggravate local tissue damage through the production of autoantibodies (Zhang et al., 2022; Zhou et al., 2024c; Liu et al., 2018).

It was demonstrated that the density of monocytes/macrophages and dendritic cells remained dynamically stable in the absence of inflammation. They continued to maintain their protective functions and did not exhibit a significant correlation with the inflammatory response of the mammary tissue lobule. However, once the mammary lobule becomes inflamed, the number of adaptive immune cells increases significantly (Degnim et al., 2014). Ruffel et al. further noted that a variety of immune cells are present in normal breast tissue. These cells monitor the immune microenvironment, are associated with chronic inflammatory responses, and can recognise novel antigens (Pal et al., 2021). In summary, the immune microenvironment of PCM constitutes a complex network initiated by DAMPs and molded by the interaction between innate and adaptive immune responses. Its fundamental characteristics include the imbalance between pro-inflammatory and anti-inflammatory responses, as well as the abnormal differentiation and infiltration of plasma cells.

## 3 Roles of innate immune cells in PCM

### 3.1 Macrophages

A study found that M2 macrophages are highly expressed in PCM and GLM tissues (Kong et al., 2020). The study also found that M2 macrophage expression was associated with the disease stage of GLM; however, no similar changes were observed in PCM. A single-cell omics study revealed marked macrophage infiltration within the local lesion tissues of patients with PCM, accompanied by enhanced signaling interactions from macrophages to B lymphocytes (Ni et al., 2025). M1 macrophages can secrete massive pro-inflammatory cytokines, such as interleukin-2 (IL-2), interleukin-6 (IL-6), interleukin-12 (IL-12), TNF-α, *etc.*, and then recruit more immune cells to drive the inflammatory process strongly (Zhou et al., 2024b). In the later stages of inflammation, macrophages usually polarize into M2 subtypes. Then they can serve a phagocytic function and promote inflammation reduction and tissue repair by secreting anti-inflammatory factors, like interleukin-10 (IL-10) and transforming growth factor-beta (TGF-β) (Shapouri-Moghaddam et al., 2018; Yunna et al., 2020). However, persistent activation of M1 macrophages may exacerbate tissue damage (Shapouri-Moghaddam et al., 2018; Fu et al., 2023). This process may be one of the key mechanisms underlying chronicity and tissue destruction in PCM. In the process of GLM, macrophages are key participants in granuloma formation (Yuan et al., 2022; Krausgruber et al., 2023). Some mechanisms by which herbal medicine can treat GLM may have great relationships with macrophages; they may inhibit the chemokine ligand-5 (CCL-5) secreted by macrophages to relieve the inflammatory response (Wang et al., 2018).

Despite initial insights into macrophage polarization in GLM, research on its link to PCM is scarce, likely due to PCM’s pathology and limited animal models. Macrophages may play a significant role in PCM’s development, persistence, and remission. Future research into these mechanisms could be crucial for understanding PCM’s pathogenesis.

### 3.2 DCs

A study conducted single-cell RNA sequencing on bovine peripheral blood mononuclear cells and found that DCs were present in the mammary ductal epithelium. Their activation played a major role in the development of inflammation (Gao et al., 2022). Many studies have found that, during an inflammatory response, DCs can migrate to the site of inflammation and initiate protective immunity to participate in immune maintenance and promote inflammation regression (Liu J. et al., 2023; Worbs et al., 2017). Research on PCM’s function and mechanisms is scarce, mainly focusing on immune defense and maintenance. There’s a need to investigate how DC differentiation and antigen presentation affect PCM-related changes, like breast ductal dilatation. Future studies on DCs could reveal key immune mechanisms in PCM disease.

### 3.3 Neutrophils

Studies about the relationship between neutrophils and breast diseases now include: (1) breast cancer: the inflammatory microenvironment caused by the excessive recruitment of neutrophils and the formation of NETs may promote the metastasis of tumors (Xiao et al., 2021; Wang et al., 2024; Tyagi et al., 2021; Park et al., 2016; He et al., 2024). In addition, it has been found that NETs also affect chemoresistance (Mousset et al., 2023; Xie et al., 2024); (2) benign breast diseases: studies mainly focus on bacterial infections, for example, inhibiting the neutrophil infiltration can relief mastitis (Hu et al., 2019); (3) PCM: current studies are mostly based on blood markers (e.g., neutrophil/lymphocyte ratio NLR) to aid diagnosis (Zheng Y. et al., 2022). However, PCM may be accompanied by bacterial infection only when it forms a local abscess at a later stage of the disease. Therefore, the role of neutrophils still needs further exploration, especially in early PCM.

Activated neutrophils have a longer lifetime; they can participate in tissue inflammatory response and later swallow damaged cells to promote the regression of inflammation. Some tumor-associated neutrophils exhibited stronger tumor-killing and pro-inflammatory effects (Fridlender et al., 2009), while some subtypes have potent anti-inflammatory effects (Tsuda et al., 2004). The different subtypes can also be transformed by some specific cytokines or natural killer T cells (NKT) (De Santo et al., 2010). Certainly, there is a perspective that attributes the varying effects to distinct cytokines secreted by different subtypes, which may facilitate macrophage polarization into M1 or M2 phenotypes, thereby regulating the direction of the inflammatory response (Sica and Mantovani, 2012). Hypothesis based on aseptic liver injury models (Mathias et al., 2006; Wang et al., 2017): Neutrophils may undergo reverse migration from PCM lesions back to circulation, potentially limiting tissue damage. Whether this mechanism operates in PCM remains to be determined. This physiological mechanism can retain a substantial number of neutrophils within the body, thereby mitigating tissue damage caused by excessive neutrophil recruitment and activation. Furthermore, it aids in preserving the immune homeostasis (Liew and Kubes, 2019). NETs possess a substantial DNA reticular framework composed of histones and antimicrobial proteins. These structures are capable of interacting with various pattern recognition receptors, such as TLRs, complement receptors and chemokine receptors, thereby participating in immunomodulation under diverse stimulating conditions both *in vitro* and *in vivo* (Li et al., 2023). Furthermore, this reaction may occur more rapidly under bacterial stimulation (Pilsczek et al., 2010; Brinkmann et al., 2004), but its effects on aseptic inflammation remain unclear.

### 3.4 Natural killer cell

**FIGURE 1:**
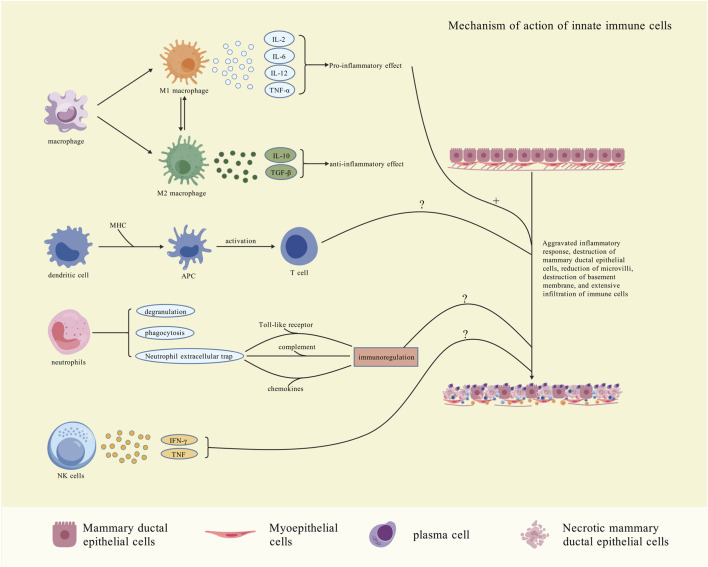
Mechanism of action of innate immunity cells. Macrophages, DCs, neutrophils, and NK cells form the primary defense against stimuli like trauma. Early in the disease, macrophages polarize into M1, secreting pro-inflammatory cytokines (IL-2, IL-6, IL-12, TNF-α). Later, they shift to M2, releasing anti-inflammatory factors (IL-10, TGF-β) to resolve inflammation and promote tissue healing and repair. DCs bind antigens to form APCs *via* the Major Histocompatibility Complex (MHC), promoting T cell activation. Neutrophils are activated by TLRs, the complement system, and chemokines, and modulate immunity. Their role in PCM progression and pathology is unclear. NK cells release IFN-γ and TNF to kill damaged cells and promote inflammation, but their exact mechanism in PCM remains unknown [Created with BioGDP.com (Jiang et al., 2025)].

NK cells demonstrate elevated expression levels in GLM and PCM compared to normal breast tissue. The quantity of NK cells could serve as a crucial biomarker for assessing the stages of the GLM process. (Kong et al., 2020). This discovery was also confirmed in IGM (Emsen et al., 2021). Different subtypes of NK cells fulfill various roles in breast diseases. For example, certain tumor-promoting immature NK cells can activate the Wnt signaling pathway, which in turn activates cancer stem cells and promotes the progression of triple-negative breast cancer (TNBC) (Thacker et al., 2023). Studies on NK cells and PCM are rare, and there’s no evidence of significant changes in NK cells across PCM stages. Because of the different impacts of NK cell subtypes, their role in PCM progression needs further study (Figure 1).

## 4 Roles of adaptive immune cells in PCM

### 4.1 T lymphocytes

#### 4.1.1 Th cells

The existence of Th cells has been demonstrated in benign breast tissue diseases (Cabioglu et al., 2023). A study discovered that the ratio of Th cells in the patient’s peripheral blood was lower than that in healthy individuals; this may be associated with the tissue collection of IGM (Emsen et al., 2021). Th cells play a crucial role in the inflammatory reaction and immune response of breast tissues.

##### 4.1.1.1 Th1 cells

Th1 cells predominantly secrete IFN-γ and IL-12 to augment their functional activity in APCs (Dong, 2021). A study identified elevated expression levels of both IFN-γ and IL-12A in PCM tissues (Liu et al., 2017). IL-12, recognized as a surface marker of M1 macrophages, can facilitate the differentiation of CD4^+^ T lymphocytes into Th1 cells to activate the immune response functions (Zhao et al., 2022). IFN-γ facilitates the further differentiation of Th1 cells (Afkarian et al., 2002; Martín-Fontecha et al., 2004). It synergizes with TNF-α to activate the Janus Kinase and Signal Transducer and Activator of Transcription associated signaling pathway (JAK/STAT1/IRF1), thereby inducing the release of carbon monoxide (CO) and promoting cell. A study discovered that the inflammatory response could be mitigated through the utilization of neutralizing antibodies both (Karki et al., 2021; Jin et al., 2024). IFN-γ can stimulate macrophages to polarize towards M1 macrophages. Furthermore, IFN-γ may also enhance TNF-α release, thereby exacerbating the inflammatory response and tissue damage (Shapouri-Moghaddam et al., 2018; Zhao et al., 2022). Importantly, in certain non-bacterial inflammatory and autoimmune diseases, the condition may progress to chronic inflammation if the acute inflammatory response, precipitated by alterations in tissue microenvironments and trauma-related factors, cannot be effectively managed or removed. During this progression, the persistent antigens continuously stimulate an inflammatory response. Subsequently, adaptive immunity can be augmented and specialized, leading to the production of a variety of pro-inflammatory factors and the formation of positive feedback mechanisms for regulation. Nevertheless, persistent pro-inflammatory environments may reversely induce the aging and hypo-responsiveness of T lymphocytes (Moro-García et al., 2018; Goronzy and Weyand, 2017). This perspective has been substantiated in the study investigating the efficacy of ozone in refractory GLM, as IFN-γ is capable of ameliorating inflammation (Cabioglu et al., 2023). This phenomenon may be associated with decreased Th1 cell reactivity and cell depletion in refractory GLM, with no pertinent research available in PCM. Furthermore, dysregulated Th1 cell activation represents a plausible mechanism for both disease initiation and inflammatory amplification (Sun et al., 2024a). Furthermore, a study revealed that impairing the differentiation of Th1 cells may subsequently contribute to the alleviation of colitis and related allergic reactions (Chandra et al., 2023). Regrettably, its role in PCM remains uncertain.

##### 4.1.1.2 Th2 cells

Th2 cells are capable of secreting IL-4, interleukin-5 (IL-5), and IL-13 (Nakayama et al., 2017). The activation of Th2 cells constitutes a positive feedback mechanism (Zhu, 2015). Th2 cells are also capable of secreting certain cytokines that inhibit the differentiation of Th1 cells and then promote the differentiation of B lymphocytes. Furthermore, they can selectively activate macrophages, inducing their polarization into the M2 phenotype. Th2 effector cells can be activated within lymph nodes and subsequently migrate to inflammatory tissues to exert functions (Ruterbusch et al., 2020).

Studies found that the levels of IL-4 in IGM patients were not significantly different from those in normal patients; however, other inflammatory cytokines such as interleukin-8 (IL-8) and IL-10 showed significant differences increased (Koksal et al., 2020). It has been established that IL-4 functions to stimulate the proliferation of B lymphocytes as early as 1982 (Howard et al., 1982). A clinical study constructed a knowledge graph related to traditional Chinese medicine. Based on this knowledge graph, the study identified a compound prescription consisting of eight herbs: Fructus forsythiae, Herba violae, Uniflower swisscentaury root, Danshen, Astragalus, Taraxacum, Liquorice, and Honeysuckle. This prescription was used to treat patients with PCM. The findings indicated that, in comparison to Western medicine methylprednisolone, the Chinese herbal formulation demonstrated superior efficacy and a lower recurrence rate. Moreover, serum levels of IL-2, IL-4, IL-6, IFN-γ, interleukin-1β (IL-1β), and TNF-α were markedly diminished in the group receiving Chinese herbal treatment medicine (Liu C. et al., 2023). Research focused on breast microecology and metabolomics has also demonstrated that the traditional Chinese medicine compound Yanghe-Tang exhibits superior efficacy in the treatment of PCM. During this process, the expression of IL-4 and IL-6 in the patients also decreased obviously (Ma et al., 2025). However, this study observed an increase in the levels of IgA, IgM, and IgG in patients within the treatment group receiving the Chinese medicine compound. This variance may be attributable to differing activation statuses of downstream pathways and the various stages involved in PCM. Metabolomics analysis revealed that Yanghe-Tang modulates key inflammatory metabolites (such as 9,10-epoxyoctadecenoic acid, 4,5-dihydroorotic acid, glycerophosphocholine, and arachidic acid). Studies have also demonstrated that IL-4 can induce B lymphocytes to synthesize IgE as well as a small quantity of IgG4 (Pène et al., 1988). And it is associated with the proliferation of B lymphocytes in immature human fetuses, as well as the Ig isotype conversion (Punnonen and de Vries, 1994). A study utilized alpha-galactosylceramide to activate NKT cells, resulting in the promotion of IL-4 secretion. This activation could subsequently stimulate Th2 cells and inhibit the differentiation of Th1 cells, thereby promoting Th2-like responses (Sharif et al., 2001). Furthermore, IL-4 possesses the ability to activate macrophages *via* exosomes, inducing their polarization towards the M2 phenotype. This process subsequently enhances fat metabolism and mitochondrial activation, thereby exerting anti-inflammatory effects (Phu et al., 2022). In specific rheumatic autoimmune diseases, it is incontrovertible that IL-4 plays a significant role (Chen et al., 2019; Iwaszko et al., 2021). Although the downstream pathways and IL-4 activation mechanisms are not fully understood, IL-4 plays a significant role in PCM-related biological processes. The primary characteristics and pathogenesis of PCM involve excessive activation and differentiation of B lymphocytes into plasma cells. The infiltration of plasma cells into mammary ducts and decreased IL-4 levels may slow PCM progression by suppressing B lymphocyte activation. However, the specific effects of IL-4 on PCM require further investigation.

IL-5, which facilitates eosinophil lineage proliferation through transport amplification pathways (Jorssen et al., 2024), is closely associated with type 2 inflammatory responses (Gandhi et al., 2016). A study examined respiratory diseases exacerbated by aspirin and concluded that IL-5 may influence the proliferation and differentiation of B lymphocytes into plasma cells. Therefore, inhibiting the interaction between IL-5 and IL-5R could potentially decrease plasma cell infiltration in respiratory tissues extent (Buchhei et al., 2020). The activation effects of IL-5 on plasma cells were also demonstrated previously in 2008 (Emslie et al., 2008). Nevertheless, regarding benign breast diseases, particularly PCM, research concerning IL-5 is exceedingly scarce. This may be attributed to the fact that the function of eosinophils in PCM remains inadequately understood, necessitating further investigation in the future.

IL-13 has previously been recognized as a pivotal regulator of allergic inflammatory responses; it exhibits similar functions to IL-4, as they share a common receptor subunit, specifically the alpha subunit of IL-4R (Hershey, 2003). IL-13 may function to stimulate the proliferation of B lymphocytes (McKenzie et al., 1993). This function may promote the production of plasma cells and aggravate local plasma cell infiltration in PCM. Furthermore, it can also induce the phosphorylation of STAT6, thereby facilitating macrophage polarization towards the M2 phenotype (Rahal et al., 2018). Most contemporary research on IL-13 concentrates on atopic dermatitis and associated conditions. Lebrikizumab demonstrates its therapeutic efficacy by specifically targeting IL-13 (Jandová et al., 2025; Abraheem et al., 2025; Mitroi et al., 2024). An increasing number of studies suggest that IL-13 is a pro-inflammatory cytokine primarily impacting epithelial cells, smooth muscle cells, and similar cell types. Additionally, it has the potential to exacerbate eosinophilic airway inflammation (Nakagome and Nagata, 2024). However, research regarding the effects of IL-13 on PCM remains scarce. We anticipate that this may emerge as a new area of investigation into the roles of IL-4 and IL-13 in the pathogenesis of PCM.

In cases of benign breast diseases, a study compared serum interleukin-33 (IL-33) levels among a healthy population, patients with GLM, and breast cancer patients. No statistically significant difference was observed; however, serum IL-33 levels in patients with GLM and breast cancer were higher than those in the healthy group population (Haghbin et al., 2023). However, another study found that IL-33 levels in the serum of IGM patients were significantly higher than those in breast cancer patients (Yigitbasi et al., 2017). This might serve as one of the indicators of benign breast diseases. IL-33 is a member of the Interleukin-1 (IL-1) family. It can be released in response to tissue damage; however, it is not the cytokine secreted by Th2 cells. IL-33 can promote the maturation of eosinophils (Johnston and Bryce, 2017) and regulate basophil granulocytes (Valent, 2009). Furthermore, it can also augment both antigen-dependent and antigen-independent T cell responses. In conjunction with IL-12, it stimulates NK cells and NKT cells to produce IFN-γ (Smithgall et al., 2008). Since it is an alarm protein produced in response to tissue damage, necrosis, and similar conditions, and is scarcely secreted by live cells under steady conditions, studies suggest it is a form of DAMP (Fournié and Poupot, 2018). It can bind to the ST2 receptor, which is expressed on the surface of Th2 cells, thereby stimulating Th2 cell activation and promoting the secretion of anti-inflammatory cytokines such as IL-4, IL-5, and IL-13 (Lukens et al., 2012). Perhaps this is associated with various stages of disease. Current research suggests that the IL-33/ST2 axis can activate DCs, macrophages, and neutrophils (Günther et al., 2017; Liew et al., 2016). And its secretion is not restricted by the level of differentiation of Th2 cells. Recent studies have demonstrated that IL-33 can promote macrophage polarization towards the M2 phenotype and facilitate the resolution of inflammation through metabolic reprogramming. This process may be associated with the role of IL-33 in response to cellular stimuli death (Faas et al., 2021). Nevertheless, research concerning IL-33 in PCM remains scarce; however, it may develop into a new area of study in the pathogenesis and treatment of PCM.

##### 4.1.1.3 Th17 cells

TGF-β and IL-6 facilitate the polarization of naïve T lymphocytes towards Th17 cell subtypes. Th17 cells are capable of secreting specific cytokines, namely, interleukin-17 (IL-17) and a modest amount of IFN-γ. A domestic study has identified that the serum levels of IL-17 in PCM patients are significantly elevated compared to healthy women. Furthermore, IL-17 levels are correlated with various clinical stages of PCM, with higher levels observed in the acute and abscess phases relative to other stages. IL-17 is involved in neutrophil proliferation, maturation, migration, and other processes. Furthermore, it can induce a substantial production of pro-inflammatory cytokines such as IL-6, IL-8, and TNF. Additionally, it contributes to promoting granulopoiesis and the maturation of DCs (Nakae et al., 2002).

A study investigated the serum levels of IL-17 in patients with IGM and observed that these levels were elevated in IGM patients compared to healthy individuals (Koksal et al., 2020). Another study determined that the levels of interleukin-23 (IL-23) and interleukin-22 (IL-22) in IGM patients exhibited statistically significant differences compared to the healthy control group, whereas no difference was observed in IL-17 levels (Saydam et al., 2021). The disparate outcomes observed in these two studies may be attributable to the differing stages of IGM patients. Nonetheless, they all suggest that Th17 cells and their associated cytokines are significant in benign breast diseases, particularly in non-lactating mastitis. A case-control study also examined the disparity in cytokine levels between breast tissues from PCM patients and paraneoplastic normal tissues in other benign breast diseases. Unfortunately, they did not observe a statistically significant difference in IL-17A levels between the two (Liu et al., 2017). As the majority of specimens in this study were already at advanced stages of PCM abscess development, it was not feasible to determine whether Th17 cells contributed to PCM pathogenesis. Although current research on the mechanisms underlying the relationship between PCM and IL-17 remains somewhat unclear, the pro-inflammatory role of IL-17 is an indisputable fact. Further investigation into the role of IL-17 in the progression of PCM is necessary in future studies.

##### 4.1.1.4 Tfh cells

Tfh cells predominantly reside within lymph nodes or the spleen. They play a crucial role in promoting the maturation of B lymphocytes, antibody class recombination, hair growth center formation, and the production of memory B lymphocytes in the bloodstream (Shekhar and Yang, 2012). Tfh cells are capable of producing interleukin-21 (IL-21), C-X-C chemokine receptor-5 (CXCR-5), and Programmed Death-1 (PD-1). They facilitate the differentiation of B lymphocytes within the hair growth center into plasma cells or memory B cells (Crotty, 2014). Furthermore, they can induce increased glycolysis (Sharabi et al., 2020). A study used baicalin from Chinese herbs to treat lupus nephritis in mice. It inhibited Tfh cell proliferation and lowered IL-21 levels, showing anti-inflammatory effects (Yang et al., 2019). A study additionally discovered that inhibiting the differentiation of Tfh cells may contribute to a reduction in colonic inflammation (Zhang et al., 2019). The preceding statement indicates that Tfh cells might serve a pro-inflammatory function in the advancement of inflammation and are intrinsically linked to plasma cell production. The role of Tfh cells in benign breast diseases is unclear, and no studies have explored their link to PCM pathogenesis. Further research is needed to see if Tfh cells influence benign breast progression diseases.

#### 4.1.2 Treg cells

**FIGURE 2:**
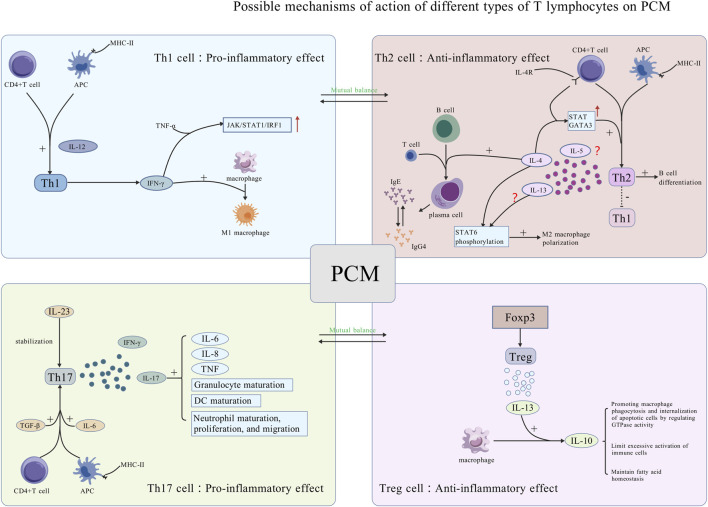
Potential mechanisms of action of various types of T lymphocytes on PCM. Th1 and Th2 cells have opposing functions and regulate each other. Th1 cells promote inflammation by secreting IFN-γ, activating the JAK pathway, and promoting macrophage polarization into M1 macrophages, thus playing a pro-inflammatory role. Th2 cells inhibit Th1 differentiation and secrete cytokines that promote B lymphocyte maturation into plasma cells, exacerbating local plasma cell infiltration of PCM. Th17 and Treg cells have opposite functions and regulate each other. Th17 cells are activated by TGF-β and IL-6, stabilized by IL-23, and secrete IL-17 and IFN-γ, promoting cytokine secretion and cell maturation. Treg cells, activated by Foxp3, secrete IL-13, encouraging IL-10 production by macrophages for anti-inflammatory effects [Created with BioGDP.com (Jiang et al., 2025)].

Treg cells are a type of immunomodulatory cell governed by the transcription factor Foxp3. A study isolated CD4^+^ T lymphocytes from breast tissues of NPM patients. It observed a decrease in the levels of Treg cells, which exert anti-inflammatory effects, while the levels of Th17 cells, which exert pro-inflammatory effects, increased. The disruption of this balance may be a major mechanism in the onset of NPM and its prolonged progression (Zhao et al., 2023). However, this study did not differentiate GLM and PCM for independent analysis. Furthermore, research has demonstrated that Treg cells can induce macrophages to secrete IL-10 through the secretion of IL-13. Subsequently, IL-10 can modulate the autocrine-paracrine pathways of GTPases, thereby enhancing the phagocytosis and internalization of apoptotic cells by macrophages, which in turn promotes the regression of inflammation (Proto et al., 2018). Furthermore, IL-10 possesses intrinsic anti-inflammatory properties; it mitigates the overactivation of immune cells and the secretion of pro-inflammatory cytokines. Additionally, it plays a role in maintaining the immune equilibrium and facilitating the resolution of inflammation by regulating fatty acid homeostasis (York et al., 2024). Although the regulatory mechanisms underlying the transcriptional properties of Treg cells are not fully understood, their anti-inflammatory function has been substantiated by numerous studies (Ding et al., 2022). Focusing on benign breast diseases, a prior study employed flow cytometry to analyze the expression of Foxp3 on Treg cells across various disease stages in IGM patients. The study found that Foxp3 expression decreased during both active and remission phases when compared to the normal control group (Ucaryilmaz et al., 2022). This conclusion has also been corroborated by another study, which determined that the levels of Treg cells in the serum of IGM patients were markedly lower than those in the healthy population (Dogan et al., 2023). A study employing the two-sample Mendelian randomization methodology indicated that an elevation in Treg cell-associated cytokines could be linked to a reduced risk of mastitis (Chen et al., 2024a). Although the mastitis case data incorporated in this study were not exclusively limited to PCM, the influence of Treg cells on the breast microenvironment and the progression of inflammation remained significant important. The mechanism of ozone therapy for IGM is also associated with an increase in the levels of Treg cells (Cabioglu et al., 2023). Future research should focus on elucidating the specific mechanisms by which Treg cells contribute to the pathogenesis and progression of PCM (Figure 2).

#### 4.1.3 CTLs

CTLs induce apoptosis through the secretion of perforin and granzyme. Furthermore, the Fas ligand (FasL) is expressed on the surface of CTLs; it can bind to the Fas receptor on the target cell’s surface, subsequently activating the apoptosis signaling pathway. CTLs are significant effector cells involved in initiating apoptosis and inflammatory responses, while memory CD8^+^ T lymphocytes enable to respond rapidly upon subsequent encounters with the same antigen (Harty et al., 2000). CTLs can also secrete pro-inflammatory cytokines such as IFN-γ and TNF-α, which may exacerbate autoimmune and inflammatory responses. A study observed no significant statistical difference in the levels of CD8^+^ T lymphocytes in peripheral blood between patients with GLM and healthy individuals (Xie et al., 2025). Nevertheless, another study identified the widespread infiltration of CD8 lymphocytes within the pathological tissues of IGM (Deng et al., 2017). This phenomenon may be associated with various stages of the disease and could represent a distinguishing feature between IGM and GLM. Although a study indicated that the levels of CD8^+^ T lymphocytes in the pathological tissues of PCM were lower than those in GLM, this difference was not statistically significant (Zhou et al., 2024c). Furthermore, it is unfortunate that the study solely focused on variations among different disease types in NPM. It did not include a comparison with a healthy population, thereby rendering it difficult to determine whether CD8^+^ T lymphocytes infiltrate PCM lesions. The investigation into ozone therapy for refractory IGM also revealed that the levels of TNF-α and CD8^+^ T lymphocytes secreted into the peripheral blood of patients with refractory IGM significantly increased following ozone treatment (Cabioglu et al., 2023). This phenomenon may be associated with the depletion and diminished cellular reactivity of CD8^+^ T lymphocytes induced by the prolonged and repeated antigenic exposure stimulation (Zhang B. et al., 2024). Hence, the depletion of CD8^+^ T lymphocytes should be taken into account when investigating the pathogenesis of recurrent conditions PCM. A study employed the transmission electron microscope and determined that the pathogenesis of GLM is associated with an inflammatory response induced by cellular pyroptosis. This process involves cysteinyl aspartate-specific protease-1 (caspase-1) (Zuo et al., 2021), a member of the caspase family similar to caspase-8, which is activated during apoptosis induction by CTLs through the Fas pathway. Caspase-1 is associated with apoptosis and autophagy. Despite this, the current understanding of whether the caspase family contributes to PCM and the specific mechanisms involved remains incomplete. Nevertheless, the role of CTLs in PCM and the underlying mechanisms continue to represent a promising direction for future research.

### 4.2 B lymphocytes

**FIGURE 3:**
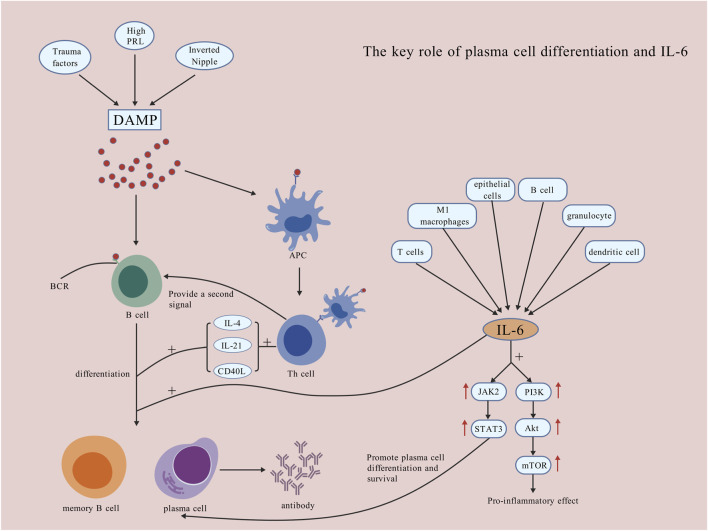
The key role of plasma cell differentiation and IL-6. DAMP mode activates B lymphocytes, providing antigenic signals that promote their differentiation into plasma cells and antibody production, which worsens local damage. IL-6, produced by T lymphocytes, M1 macrophages, epithelial cells, B lymphocytes, granulocytes, and DCs, activates the JAK2/STAT3 pathway to promote plasma cell survival and differentiation, and the PI3K/Akt/mTOR pathway for inflammation. IL-6 also directly promotes B lymphocyte differentiation into memory B lymphocytes and plasma cells [Created with BioGDP.com (Jiang et al., 2025)].

An essential pathological hallmark of PCM is the extensive differentiation and aggregation of plasma cells (Zhang et al., 2022). B lymphocytes can differentiate into plasma cells and subsequently produce antibodies that erroneously target their own tissues. A study employed immunohistochemical techniques and observed the overexpression of CD20^+^ B lymphocytes in tissue of PCM (Zhou et al., 2024c). Furthermore, the substantial accumulation of plasma cells within breast tissue consistently exacerbated the local inflammatory responses to certain stimuli extent. A study established that the infiltration of plasma cells in PCM tissues of mice administered with the anti-inflammatory agent, sinomenine hydrochloride, diminished and exhibited a dose-dependent relationship (Liu et al., 2020). Despite the over-differentiation and activation of plasma cells, their anti-apoptotic properties may also contribute to the recurrent and prolonged nature of PCM. This characteristic is closely linked with B-cell lymphoma-2 (Bcl-2), a gene that suppresses apoptosis. Plasma cells are among the target cells of Bcl-2. Elevated levels of Bcl-2 protein can enhance the anti-apoptotic properties of plasma cells, potentially leading to a range of plasma cell-related diseases through mechanisms such as calcium conduction pathways (Vervliet et al., 2016; Alvanidis et al., 2025). A study revealed that in successfully induced mouse PCM models, the levels of Bcl-2 protein in lesion tissues increased (Liu et al., 2018). Over-activated plasma cells secrete large amounts of immunoglobulins like IgG and IgM, potentially attacking tissues and triggering a resistant inflammatory cycle healing. Nevertheless, current research concerning the specific antibody types present in the lesion tissues of PCM, as well as potential category shifts among these antibodies, remains scarce. Furthermore, the mechanisms by which plasma cells influence PCM, the detailed pathways of relevant immune signaling, and the interactions between T cells and plasma cells constitute areas that require further investigation. These aspects should be explored more profoundly in future studies (Figure 3).

## 5 Potential targets for immunotherapy of PCM

### 5.1 The balance of M1/M2, Th1/Th2 and Th17/Treg

The immune equilibrium and inflammatory responses are contingent upon the balance among various polarization states in macrophages, referred to as the M1/M2 balance. A study discovered that macrophages have the capacity to transition between various polarization states. In our prior description, there was a significant expression of M2 macrophages in the tissues of PCM (Kong et al., 2020). A study discovered that the polarization of M1 macrophages may contribute to the progression of inflammation (Ji et al., 2023), whereas inhibiting M1 macrophages or encouraging their transformation into M2 macrophages facilitated a reduction in inflammatory responses (Wang et al., 2018; Cai et al., 2020; Van den Bossche et al., 2016; Yu et al., 2024). Although it is challenging to determine whether a difference exists in the M1/M2 ratio between tissues in PCM and healthy populations, we can hypothesize the following. Regardless of the pro-inflammatory or anti-inflammatory effects exerted by various macrophage polarization states, the disruption of this balance may constitute one of the mechanisms underlying persistent inflammatory responses in PCM lesions. Furthermore, this ratio could serve as a prognostic indicator for the progression of PCM. In future research, the modulation of this balance may become a primary focus in the treatment of PCM.

It is analogous to the M1/M2 balance, wherein Th1 and Th2 cells primarily have opposing roles in the inflammatory process response. A study employed flow cytometry to isolate CD4^+^ T lymphocytes in NPM lesion tissues and observed an increase in pro-inflammatory cytokine expression alongside a decrease in anti-inflammatory cytokine expression. This finding suggests a disruption in Th1/Th2 immune balance (Zhao et al., 2023). This imbalance has also been demonstrated in other studies or the pathogenesis of GLM (Zheng B. et al., 2022). It is anticipated that the inflammatory regression of PCM may be facilitated through the regulation of the Th1/Th2 balance in the future.

Th17 cells and Treg cells constitute a pair of cell types that mutually regulate one another and predominantly exert antagonistic effects in the inflammatory response. An imbalance of Th17/Treg has also been observed in lesion tissues NPM (Zhao et al., 2023). Different signal pathways may cause the shift in Th17/Treg balance and subsequently trigger autoimmune diseases (Lee, 2018; Chen et al., 2024b). Current research concerning the imbalance of Th17/Treg in PCM and its potential mechanisms remains scarce and warrants further investigation in future studies (Figure 2).

### 5.2 Complement system

**FIGURE 4:**
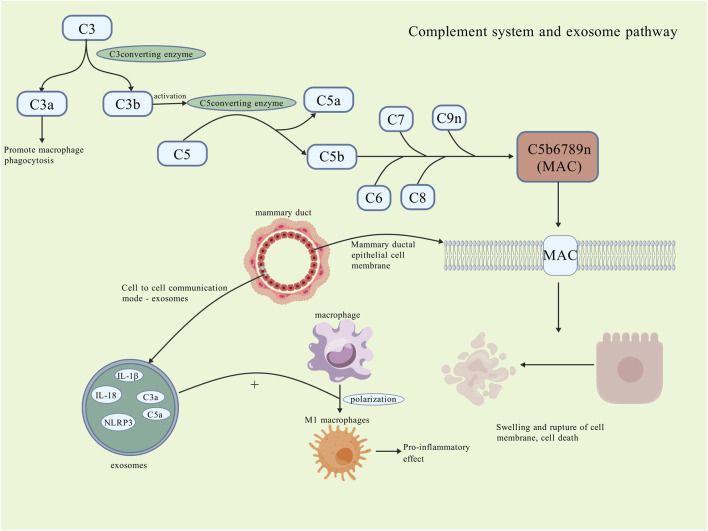
Complement system and exosome pathway. The complement system activates in the PCM microenvironment, where C3 cleaves into C3a and C3b; C3a promotes macrophage phagocytosis, while C3b activates C5 convertase, cleaving C5 into C5a and C5b. C5b, along with C6-C9, forms the MAC that inserts into the cell membrane, destroying it and causing swelling and rupture of breast ductal epithelial cells, worsening local damage. Exosomes, a novel intercellular communication form, also contribute to cell necrosis by carrying cytokines and inflammatory vesicles, promoting macrophage polarization to M1 and pro-inflammatory effects. The specific roles of the complement system and exosomes remain unclear [Created with BioGDP.com (Jiang et al., 2025)].

A study observed that the levels of C3/C3a, C3a receptors (C3aR), C5/C5a, and C5a receptor 1 (C5aR1) were elevated in the lesion tissues of PCM and GM. It suggests activation of the complement system during the pathogenesis of PCM (Li X. Q. et al., 2022). Inflammatory factors mediated by C3 have also been regarded as a contributing factor in the pathogenesis of GLM (Xie et al., 2025). A study identified the presence of MAC in the cell membrane of mammary ductal epithelial cells; additionally, epithelial cell injury and apoptosis were observed in the lesion tissues of PCM. This process may be mediated by MAC (Zhang et al., 2022). All of these observations indicate that activation of the complement system may contribute to the exacerbation of inflammation in PCM. Therefore, further research is needed in the future to investigate the role of the complement system in the course of PCM (Figure 4).

### 5.3 Exosomes

Exosomes have the capacity to transfer various substances, such as proteins, lipids, and others, to recipient cells, thereby eliciting specific immunomodulatory effects or inducing inflammation responses (Chan et al., 2019). A study discovered that exosomes derived from mammary epithelial cells have the capacity to activate downstream pathways and promote the polarization of macrophages towards the pro-inflammatory M1 subtype, thereby exacerbating local inflammation responses (Ji et al., 2023; Cai et al., 2020). In the aforementioned study of the complement system, it was observed that although there was no significant difference in the expression of C3a and C5a in the serum of PCM patients. However, these proteins were highly expressed in the exosomes. This phenomenon may be associated with the development and dissemination of the inflammation (Li X. Q. et al., 2022). Furthermore, a substantial expression of MAC in exosomes was also identified in PCM patients (Zhang et al., 2022). A study inhibited the secretion of exosomes and observed that the local inflammatory cell infiltration was significantly attenuated in mouse models of PCM (Wang et al., 2019). A similar phenomenon was also identified in research pertaining to general inflammatory diseases of the breast (Lu et al., 2023). A study also indicated that the expression of the inflammatory vesicle NLRP3 in lesion tissues of PCM mouse models was significantly elevated. Conversely, the expression of the pro-inflammatory factor IL-1β was notably downregulated following inhibition NLRP3 (Sun et al., 2024b). However, this study does not examine whether the relationship between this inflammatory vesicle and exosomes extends to PCM. The involvement of exosomes in macrophage polarization and complement regulation suggests this intercellular communication could be a promising research avenue for PCM pathogenesis and therapy (Figure 4).

### 5.4 Circadian rhythms of immune cells

In recent years, circadian rhythms have emerged as a prominent area of research; the biological clock plays a significant regulatory role in the functioning of nearly all tissues and organs. Disruption of circadian rhythms may result in either hyperactivity or weakened immune responses, potentially leading to certain pathological changes (Fagiani et al., 2022). A study suggested that the process by which lymphocytes circulate to detect antigens is also aligned with circadian rhythms, which may influence the adaptive immunity (Druzd et al., 2017). Despite the fact that lymphocytes, DCs, and neutrophils also exhibit circadian rhythms [207], leukocyte homeostasis and their migration to inflamed tissues also demonstrate certain circadian patterns [208]. All of these processes are linked to clock genes. Consequently, it can be inferred that both innate and adaptive immune cells possess their own biological clocks, which play a crucial role in inflammatory responses and anti-tumor mechanisms. However, no research currently establishes a connection between this immune cell characteristic and the initiation and progression of PCM; further investigation may be required in future studies.

### 5.5 The activation of immune cytokines and signal pathways

#### 5.5.1 IL-6 and IL-6/JAK2/STAT3 signal pathway

IL-6 is a significant pro-inflammatory cytokine; its excessive secretion has the potential to exacerbate tissue inflammation and cause localized damage (Kang et al., 2019). A study determined that in mouse models of mastitis induced by lipopolysaccharide (LPS), there was a significant increase in IL-6 levels. Furthermore, the attenuation of the inflammatory response following treatment with anti-inflammatory agents was closely associated with a reduction in IL-6 levels (Lai et al., 2017). A study concerning clinical mastitis and subclinical mastitis in cattle also determined that the levels of IL-6 are significantly elevated increased (Vitenberga-Verza et al., 2022). It can be inferred that, in the pathogenesis of PCM, M1 macrophages may secrete IL-6. Subsequently, IL-6 can activate B lymphocytes, promoting their proliferation and differentiation into plasma cells that secrete large quantities of antibodies, potentially resulting in damage to normal tissues. IL-6 is involved in the activation of Th cells, encouraging their differentiation into Th17 cells. These Th17 cells can produce IL-17 and IFN-γ, which further exacerbate inflammatory responses. Moreover, IL-17 can enhance the maturation of granulocytes and DCs, as well as stimulate the secretion of IL-6, creating a positive feedback loop that amplifies local tissue inflammation damage.

IL-6 can bind with its specific receptors, subsequently activating downstream JAK2. This activation leads to the phosphorylation of the transcription activator STAT3. A study successfully established murine PCM models through the activation of the IL-6/JAK2/STAT3 signaling pathway (Liu et al., 2018). Furthermore, the aforementioned study established that IL-6 levels in the diseased tissues of the PCM mice, which were treated with the pathway inhibitor AG-490, were significantly decreased. This finding indicates that this signalling pathway played a pivotal role in the aetiology of PCM. Research indicates that the expression of IL-6 exhibits a significant positive correlation with the expression of p-STAT3 in lesion tissues of PCM patients (Liu et al., 2015). A study using network pharmacology to explore herbal combinations, Shugan-Sanjie decoction, for the treatment of PCM also demonstrated that the expression levels of IL-6 protein, p-JAK2/JAK2, and p-STAT3/STAT3 significantly increased in the model group. In contrast, these levels decreased following the intervention. Among them, active components such as quercetin, luteolin, and kaempferol exert important anti-inflammatory effects (Nan et al., 2022). Although this signalling pathway is instrumental in the differentiation and survival of plasma cells, the precise effects it exerts on the onset and various stages of PCM remain unclear. Further research into this pathway is warranted, and it may serve as a potential therapeutic target in the future.

#### 5.5.2 PI3K/Akt/mTOR signal pathway

Phosphoinositide 3-kinase (PI3K) is also a downstream cytokine of JAK (Hou et al., 2018). A study discovered that activation of the PI3K/Akt/mTOR signaling pathway contributes to the induction of inflammation and facilitates the nuclear translocation of nuclear factor kappa-B (NF-κB). It also promotes the secretion of numerous pro-inflammatory cytokines, thereby exacerbating inflammatory responses in tissues (Kim et al., 2017). A notable upregulation of p-Akt and p-mTOR in lesion tissues of PCM has been observed in a study employing immunohistochemical techniques and Western blot analysis. This finding suggests that activation of the PI3K/Akt/mTOR signaling pathway plays a significant role in the pathogenesis of PCM and may be associated with JAK activation (Wang et al., 2019). However, the specific mechanisms of the PI3K/Akt/mTOR signaling pathway and its relationship with the IL-6/JAK2/STAT3 signaling pathway, as well as NF-κB, require further investigation. It may become feasible to treat PCM by modulating these signaling pathways through immune-targeted approaches in the future.

## 6 Conclusions and outlook

As a form of aseptic inflammatory disease, the diagnosis and treatment of PCM lack specific indicators. Nonetheless, current evidence robustly supports its classification as a type of autoimmune disorder. A significant involvement of immune factors, particularly related immune cells, is evident in the pathogenesis and various stages of PCM.

Based on current research, we suggest that the DAMP mode plays an important role in the pathogenesis of PCM. Recognized factors such as trauma, hyperprolactinaemia (Peters and Schuth, 1989) and inverted nipple deformities can release DAMP, thereby activating local autoimmune responses *via* Toll-like receptors and other pathways. This process can rapidly activate the innate immune cells in mammary tissues: macrophages can polarize into the pro-inflammatory M1 subtype and subsequently secrete inflammatory factors such as IL-2, IL-6, IL-12, among others. A substantial recruitment of neutrophils occurs at lesion sites. DCs are activated and serve as vital APCs to initiate adaptive immune responses. NK cells can secrete IFN-γ and TNF-α, thereby promoting early inflammatory progression.

Subsequently, within the context of MHC-II molecules, activated APCs can present antigens, thereby facilitating the differentiation of T lymphocytes into Th cells. Among these, IL-12, secreted by M1 macrophages, plays a pivotal role in markedly promoting Th1 cell differentiation. IFN-γ secreted by Th1 cells participates in the formation of two critical positive feedback loops. Firstly, IFN-γ—along with that secreted by NK cells—further promotes the differentiation of Th1 cells. Secondly, IFN-γ also enhances the polarization of M1 macrophages. This mutually reinforcing mechanism induces a substantial increase in TNF-α levels in local tissues, thereby significantly aggravating tissue inflammation and damage. TGF-β and IL-6 within the microenvironment promote the differentiation of Th17 cells, which subsequently secrete IL-17 and IFN-γ. IL-17 not only directly exacerbates tissue damage, recruits additional immune cells (such as granulocytes), and promotes DC maturation, but also positively stimulates ongoing IL-6 secretion, thereby establishing a cycle that perpetuates an inflammatory state.

During a sustained immune response in tissues, many cytokines (such as IL-6) activate downstream signaling pathways (such as JAK2/STAT3 and PI3K/Akt/mTOR), promote NF-κB activation and secretion, and further amplify inflammatory responses. These signals simultaneously induce B lymphocytes to differentiate into plasma cells, while Th cells also participate in this process and ultimately cause plasma cells to secrete autoantibodies that attack breast tissues. The complement system is activated in this process, and its end product, MAC, then promotes the localized, dense infiltration of neutrophils and macrophages, which is closely associated with the destruction of breast ductal epithelial cells. Furthermore, intercellular communication facilitated by exosomes persistently transmits inflammatory signals and encourages the dissemination of inflammation. It is noteworthy that bacterial infections complicating the later stages of PCM are likely associated with the disruption of ductal epithelial integrity caused by the initial immune response.

Although the activation of M2 macrophages, Th2 cells, and Treg cells is theoretically indicative of the initiation of anti-inflammatory and reparative processes, in the actual pathology of PCM, this is not sufficient to reverse inflammation. Instead, the pro-inflammatory cytokine network, which is predominantly driven by M1 macrophages, Th1 cells, and Th17 cells, becomes dominant. It establishes a self-perpetuating cycle that is challenging to interrupt. These “pro-inflammatory factor storms” may result in insufficient cytokine concentrations necessary for anti-inflammatory responses or lead to lymphocyte depletion and functional impairment, thereby hindering the activation and efficacy of anti-inflammatory mechanisms effectively. In the table below, biomarkers that play critical roles in PCM lesion tissues and their potential mechanisms of action are summarized (Table 1).

**TABLE 1:** Important biomarkers and their potential mechanisms of action.

Biomarker	Expression levels in PCM	Potential mechanism	Sources of evidence
IL-4	Increased	Promote Th2 cell differentiation Promote B lymphocytes proliferation (the downstream pathway is not clear) Promote M2 macrophage polarization	Howard et al., 1982; Zhu (2015), Ma et al. (2025), Phu et al. (2022), Liu et al. (2023b)
IL-6	increased	Activate JAK2/STAT3 signaling pathway Further activate PI3K/AKT/mTOR signaling pathway (the specific mechanism is unknown) Promote Th17 cell differentiation	Ma et al. (2025), Liu et al. (2023b)
IL-12A	Increased	Promote Th1 cell differentiation Enhance antigen-presenting capacity	Liu et al., 2017; Dong (2021), Zhao et al. (2022)
IFN-γ	Increased	Promote Th1 cell differentiation Activate JAK/STAT1/IFN1 signaling pathway Promote M1 macrophage differentiation	Karki et al. (2021), Jin et al. (2024), Liu et al. (2023b)
TNF-α	Increased	Proinflammatory response	Karki et al. (2021), Liu et al. (2023b)
IL-17	Increased	Promote neutrophil proliferation Promote IL-6 secretion Promote TNF-α and secretion Promote DCs maturation	Nakae et al. (2002)
C3/C3a, C5/C5a	Increased	Formation of MAC and pro-inflammatory outcomes	Zhang et al. (2022), Li et al. (2022a)

**FIGURE 5:**
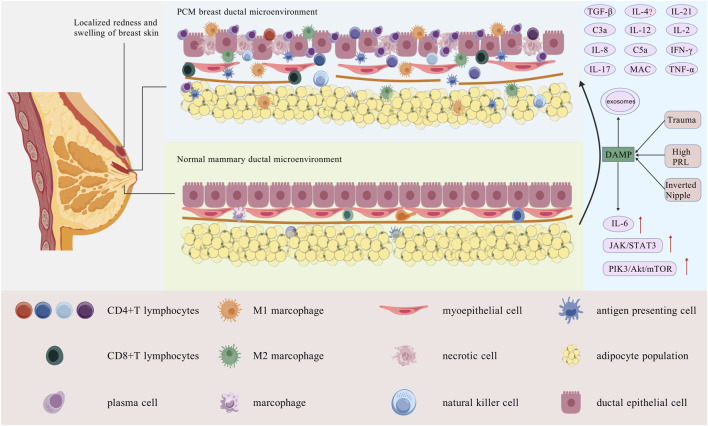
Breast duct microenvironment in normal breast tissue and PCM tissue. Local PCM presents with skin erythema, fever, and pain. Its pathology shows dilated mammary ducts with extensive plasma cell and lymphocyte infiltration. In the center, a normal mammary duct displays well-organized ductal epithelial cells, normal myoepithelial gaps, an intact basement membrane, and minimal immune cell infiltration. Traumatic injuries, hyperprolactinaemia, or nipple inversion alter the local immune microenvironment, activating DAMPs, innate immune cells, and macrophages into M1. APCs stimulate adaptive immunity, differentiating T cells and secreting cytokines, mostly pro-inflammatory, which aggravate lymphocyte infiltration and tissue damage in mammary ducts. The epithelial cells of the mammary ducts are disrupted after injury, some necrotic, with basement membrane damage, loss of integrity, and immune cell infiltration [Created with BioGDP.com (Jiang et al., 2025)].

It must be acknowledged that, as a form of non-bacterial, autoimmune inflammatory disease, the precise pathogenesis of PCM has not yet been fully elucidated. The absence of recognized animal models and the statistically significant individual differences present challenges for comprehensive research. Although evidence associates increased levels of M1 macrophages, Th1, and Th17 cells with exacerbated inflammation, the activation of M2 macrophages, Th2, and Treg cells contributes to its reduction inflammation. However, the specific mechanisms of action, inter-regulation, and equilibrium (such as the balance of M1/M2, Th1/Th2, and Th17/Treg) in the progression of PCM require further investigation. Furthermore, significant research gaps persist in numerous critical areas. The functions of neutrophils and the NETs they release remain largely unexplored. The role of the cytokine IL-4, which exhibits dual actions, is still not well understood. Tfh cells, which are essential in promoting B lymphocyte responses and antibody production, have not yet been studied in relation to the development of PCM. Additionally, the potential of exosomes as therapeutic targets requires further validation (Figure 5).

In conclusion, the current understanding of the specific mechanisms involved in the role of immunological factors in the pathogenesis of PCM remains limited. Future research should concentrate on a comprehensive analysis of the fundamental connections within the immune network to validate and enhance the aforementioned pathogenesis framework. Additionally, there should be active exploration of immune-related diagnostic markers and therapeutic targets based on this understanding, with the ultimate aim of developing more precise and efficacious therapeutic strategies PCM.

## References

[B1] Abraheem A. Sebastian N. Kreeshan F. C. Clayton T. H. Hunter H. J. A. Warren R. B. (2025). Lebrikizumab for the treatment of moderate to severe atopic eczema: real-world experience from a tertiary centre. Dermatol. Ther. (Heidelb). 10.1007/s13555-025-01614-9 41351749 PMC12872942

[B2] Afkarian M. Sedy J. R. Yang J. Jacobson N. G. Cereb N. Yang S. Y. (2002). T-bet is a STAT1-induced regulator of IL-12R expression in naïve CD4+ T cells. Nat. Immunol. 3 (6), 549–557. 10.1038/ni794 12006974

[B3] Alvanidis G. Kotsos D. Frouzaki C. Fola A. Hatjiharissi E. (2025). The potential role of BCL-2 inhibition in amyloidosis and plasma cell leukemia. Front. Oncol. 15, 1549891. 10.3389/fonc.2025.1549891 40190562 PMC11968654

[B4] Alves-Filho J. C. Sônego F. Souto F. O. Freitas A. Verri W. A. Auxiliadora-Martins M. (2010). Interleukin-33 attenuates sepsis by enhancing neutrophil influx to the site of infection. Nat. Med. 16 (6), 708–712. 10.1038/nm.2156 20473304

[B5] Ashworth M. T. Corcoran G. D. Haqqani M. T. (1985). Periductal mastitis and mammary duct ectasia in a male. Postgrad. Med. J. 61 (717), 621–623. 10.1136/pgmj.61.717.621 4040633 PMC2418337

[B6] Brinkmann V. Reichard U. Goosmann C. Fauler B. Uhlemann Y. Weiss D. S. (2004). Neutrophil extracellular traps kill bacteria. Science 303 (5663), 1532–1535. 10.1126/science.1092385 15001782

[B7] Buchheit K. M. Dwyer D. F. Ordovas-Montanes J. Katz H. R. Lewis E. Vukovic M. (2020). IL-5Rα marks nasal polyp IgG4-and IgE-expressing cells in aspirin-exacerbated respiratory disease. J. Allergy Clin. Immunol. 145 (6), 1574–1584. 10.1016/j.jaci.2020.02.035 32199912 PMC7282948

[B8] Cabioglu N. Cetin Aktas E. Emiroglu S. Tukenmez M. Ozkurt E. Muslumanoglu M. (2023). Ozone therapy restores immune dysfunction in refractory idiopathic granulomatous mastitis as a novel potential therapeutic approach. Cell Biol. Int. 47 (1), 228–237. 10.1002/cbin.11953 36378588

[B9] Cai M. Shi Y. Zheng T. Hu S. Du K. Ren A. (2020). Mammary epithelial cell derived exosomal MiR-221 mediates M1 macrophage polarization *via* SOCS1/STATs to promote inflammatory response. Int. Immunopharmacol. 83, 106493. 10.1016/j.intimp.2020.106493 32289739

[B10] Cerwenka A. Lanier L. L. (2016). Natural killer cell memory in infection, inflammation and cancer. Nat. Rev. Immunol. 16 (2), 112–123. 10.1038/nri.2015.9 26806484

[B11] Chan B. D. Wong W. Y. Lee M. M. L. Cho W. C. S. Yee B. K. Kwan Y. W. (2019). Exosomes in inflammation and inflammatory disease. Proteomics 19 (8), e1800149. 10.1002/pmic.201800149 30758141

[B12] Chandra A. Yoon S. Michieletto M. F. Goldman N. Ferrari E. K. Abedi M. (2023). Quantitative control of Ets1 dosage by a multi-enhancer hub promotes Th1 cell differentiation and protects from allergic inflammation. Immunity 56 (7), 1451–1467.e12. 10.1016/j.immuni.2023.05.004 37263273 PMC10979463

[B13] Chen Z. Bozec A. Ramming A. Schett G. (2019). Anti-inflammatory and immune-regulatory cytokines in rheumatoid arthritis. Nat. Rev. Rheumatol. 15 (1), 9–17. 10.1038/s41584-018-0109-2 30341437

[B14] Chen R. Hu B. Zhang Y. Liu C. Zhao L. Jiang Y. (2020). Differential diagnosis of plasma cell mastitis and invasive ductal carcinoma using multiparametric MRI. Gland. Surg. 9 (2), 278–290. 10.21037/gs.2020.03.30 32420252 PMC7225459

[B15] Chen J. Su B. Zhang X. Gao C. Ji Y. Xue X. (2024a). Mendelian randomization suggests causal correlations between inflammatory cytokines and immune cells with mastitis. Front. Immunol. 15, 1409545. 10.3389/fimmu.2024.1409545 39399489 PMC11466835

[B16] Chen J. Shi X. Deng Y. Dang J. Liu Y. Zhao J. (2024b). miRNA-148a-containing GMSC-derived EVs modulate Treg/Th17 balance *via* IKKB/NF-κB pathway and treat a rheumatoid arthritis model. JCI Insight 9 (10), e177841. 10.1172/jci.insight.177841 38652539 PMC11141912

[B17] Clark J. S. Bui E. Williams J. Craft B. S. (2015). Plasma-cell mastitis with two other concomitant diseases. Am. J. Med. 128 (8), e9–e10. 10.1016/j.amjmed.2015.02.021 25820163

[B18] Crotty S. (2014). T follicular helper cell differentiation, function, and roles in disease. Immunity 41 (4), 529–542. 10.1016/j.immuni.2014.10.004 25367570 PMC4223692

[B19] De Santo C. Arscott R. Booth S. Karydis I. Jones M. Asher R. (2010). Invariant NKT cells modulate the suppressive activity of IL-10-secreting neutrophils differentiated with serum amyloid A. Nat. Immunol. 11 (11), 1039–1046. 10.1038/ni.1942 20890286 PMC3001335

[B20] Degnim A. C. Brahmbhatt R. D. Radisky D. C. Hoskin T. L. Stallings-Mann M. Laudenschlager M. (2014). Immune cell quantitation in normal breast tissue lobules with and without lobulitis. Breast Cancer Res. Treat. 144 (3), 539–549. 10.1007/s10549-014-2896-8 24596048 PMC3962744

[B21] Deng J. Q. Yu L. Yang Y. Feng X. J. Sun J. Liu J. (2017). Steroids administered after vacuum-assisted biopsy in the management of idiopathic granulomatous mastitis. J. Clin. Pathol. 70 (10), 827–831. 10.1136/jclinpath-2016-204287 28931582

[B22] Ding Z. Cai T. Tang J. Sun H. Qi X. Zhang Y. (2022). Setd2 supports GATA3(+)ST2(+) thymic-derived Treg cells and suppresses intestinal inflammation. Nat. Commun. 13 (1), 7468. 10.1038/s41467-022-35250-0 36463230 PMC9719510

[B23] Dogan S. Dal F. Guler M. Sevik H. Oguz Idiz U. (2023). Is peripheral blood immunophenotyping useful to understand the etiology of Idiopathic Granulomatous? Hum. Immunol. 84 (5-7), 315–319. 10.1016/j.humimm.2023.05.001 37202243

[B24] Dong C. (2021). Cytokine regulation and function in T cells. Annu. Rev. Immunol. 39, 51–76. 10.1146/annurev-immunol-061020-053702 33428453

[B25] Druzd D. Matveeva O. Ince L. Harrison U. He W. Schmal C. (2017). Lymphocyte circadian clocks control lymph node trafficking and adaptive immune responses. Immunity 46 (1), 120–132. 10.1016/j.immuni.2016.12.011 28087238 PMC5263259

[B26] Dwyer G. K. Turnquist H. R. (2021). Untangling local pro-inflammatory, reparative, and regulatory damage-associated molecular-patterns (DAMPs) pathways to improve transplant outcomes. Front. Immunol. 12, 611910. 10.3389/fimmu.2021.611910 33708206 PMC7940545

[B27] Emsen A. Köksal H. Uçaryılmaz H. Kadoglou N. Artaç H. (2021). The alteration of lymphocyte subsets in idiopathic granulomatous mastitis. Turk J. Med. Sci. 51 (4), 1905–1911. 10.3906/sag-2012-192 33862673 PMC8569769

[B28] Emslie D. D'Costa K. Hasbold J. Metcalf D. Takatsu K. Hodgkin P. O. (2008). Oct2 enhances antibody-secreting cell differentiation through regulation of IL-5 receptor alpha chain expression on activated B cells. J. Exp. Med. 205 (2), 409–421. 10.1084/jem.20072049 18250192 PMC2271016

[B29] Faas M. Ipseiz N. Ackermann J. Culemann S. Grüneboom A. Schröder F. (2021). IL-33-induced metabolic reprogramming controls the differentiation of alternatively activated macrophages and the resolution of inflammation. Immunity 54 (11), 2531–2546.e5. 10.1016/j.immuni.2021.09.010 34644537 PMC7617137

[B30] Faccin M. Caillot O. Levêque J. Perdriger A. (2016). Plasma cell mastitis in women with rheumatoid arthritis treated with TNFα antagonists: report of 2 cases. Jt. Bone Spine 83 (5), 593–594. 10.1016/j.jbspin.2015.12.001 26774176

[B31] Fagiani F. Di Marino D. Romagnoli A. Travelli C. Voltan D. Di Cesare Mannelli L. (2022). Molecular regulations of circadian rhythm and implications for physiology and diseases. Signal Transduct. Target Ther. 7 (1), 41. 10.1038/s41392-022-00899-y 35136018 PMC8825842

[B32] Fournié J. J. Poupot M. (2018). The pro-tumorigenic IL-33 involved in Antitumor immunity: a yin and Yang cytokine. Front. Immunol. 9, 2506. 10.3389/fimmu.2018.02506 30416507 PMC6212549

[B33] Fridlender Z. G. Sun J. Kim S. Kapoor V. Cheng G. Ling L. (2009). Polarization of tumor-associated neutrophil phenotype by TGF-beta: “N1” *versus* “N2” TAN. Cancer Cell 16 (3), 183–194. 10.1016/j.ccr.2009.06.017 19732719 PMC2754404

[B34] Fu B. Xiong Y. Sha Z. Xue W. Xu B. Tan S. (2023). SEPTIN2 suppresses an IFN-γ-independent, proinflammatory macrophage activation pathway. Nat. Commun. 14 (1), 7441. 10.1038/s41467-023-43283-2 37978190 PMC10656488

[B35] Gandhi N. A. Bennett B. L. Graham N. M. H. Pirozzi G. Stahl N. Yancopoulos G. D. (2016). Targeting key proximal drivers of type 2 inflammation in disease. Nat. Rev. Drug Discov. 15 (1), 35–50. 10.1038/nrd4624 26471366

[B36] Gao Y. Li J. Cai G. Wang Y. Yang W. Li Y. (2022). Single-cell transcriptomic and chromatin accessibility analyses of dairy cattle peripheral blood mononuclear cells and their responses to lipopolysaccharide. BMC Genomics 23 (1), 338. 10.1186/s12864-022-08562-0 35501711 PMC9063233

[B37] Gollapalli V. Liao J. Dudakovic A. Sugg S. L. Scott-Conner C. E. H. Weigel R. J. (2010). Risk factors for development and recurrence of primary breast abscesses. J. Am. Coll. Surg. 211 (1), 41–48. 10.1016/j.jamcollsurg.2010.04.007 20610247

[B38] Gopalakrishnan Nair C. Hiran Jacob P. Menon R. R. Misha (2015). Inflammatory diseases of the non-lactating female breasts. Int. J. Surg. 13, 8–11. 10.1016/j.ijsu.2014.11.022 25447605

[B39] Goronzy J. J. Weyand C. M. (2017). Successful and maladaptive T cell aging. Immunity 46 (3), 364–378. 10.1016/j.immuni.2017.03.010 28329703 PMC5433436

[B40] Günther S. Deredge D. Bowers A. L. Luchini A. Bonsor D. A. Beadenkopf R. (2017). IL-1 family cytokines use distinct molecular mechanisms to signal through their shared Co-receptor. Immunity 47 (3), 510–523.e4. 10.1016/j.immuni.2017.08.004 28930661 PMC5849085

[B41] Haghbin M. Sotoodeh Jahromi A. Ranjbaran R. Abbasi M. Hashemi Tayer A. (2023). Comparison of Interleukin-33 serum levels in patients with breast cancer and idiopathic granulomatous mastitis. Asian Pac. J. Cancer Prev. 24 (5), 1629–1634. 10.31557/apjcp.2023.24.5.1629 37247282 PMC10495896

[B42] Harty J. T. Tvinnereim A. R. White D. W. (2000). CD8+ T cell effector mechanisms in resistance to infection. Annu. Rev. Immunol. 18, 275–308. 10.1146/annurev.immunol.18.1.275 10837060

[B43] He X. Y. Gao Y. Ng D. Michalopoulou E. George S. Adrover J. M. (2024). Chronic stress increases metastasis *via* neutrophil-mediated changes to the microenvironment. Cancer Cell 42 (3), 474–486.e12. 10.1016/j.ccell.2024.01.013 38402610 PMC11300849

[B44] Hershey G. K. (2003). IL-13 receptors and signaling pathways: an evolving web. J. Allergy Clin. Immunol. 111 (4), 677–690. 10.1067/mai.2003.1333 12704343

[B45] Hou Y. Wang K. Wan W. Cheng Y. Pu X. Ye X. (2018). Resveratrol provides neuroprotection by regulating the JAK2/STAT3/PI3K/AKT/mTOR pathway after stroke in rats. Genes Dis. 5 (3), 245–255. 10.1016/j.gendis.2018.06.001 30320189 PMC6176158

[B46] Howard M. Farrar J. Hilfiker M. Johnson B. Takatsu K. Hamaoka T. (1982). Identification of a T cell-derived b cell growth factor distinct from interleukin 2. J. Exp. Med. 155 (3), 914–923. 10.1084/jem.155.3.914 6977612 PMC2186613

[B47] Hu J. Huang X. (2020). Combining ultrasonography and mammography to improve diagnostic accuracy of plasma cell mastitis. J. Xray Sci. Technol. 28 (3), 555–561. 10.3233/xst-190607 32333573

[B48] Hu X. He Z. Jiang P. Wang K. Guo J. Zhao C. (2019). Neutralization of Interleukin-17A attenuates lipopolysaccharide-induced mastitis by inhibiting neutrophil infiltration and the inflammatory response. J. Interferon Cytokine Res. 39 (9), 577–584. 10.1089/jir.2019.0069 31313943

[B49] Iwaszko M. Biały S. Bogunia-Kubik K. (2021). Significance of interleukin (IL)-4 and IL-13 in inflammatory arthritis. Cells 10 (11), 3000. 10.3390/cells10113000 34831223 PMC8616130

[B50] Jandová M. Litvik R. Tichý M. Gkalpakiotis S. (2025). Atopic dermatitis successfully treated with lebrikizumab in real-world clinical practice in Czech Republic: a case series. Dermatol. Ther. (Heidelb). 10.1007/s13555-025-01608-7 41348396 PMC12872983

[B51] Ji Z. H. Gao F. Xie W. Y. Wu H. Y. Ren W. Z. Yuan B. (2023). Mammary epithelial cell-derived exosomal miR-221-3p regulates macrophage polarization by targeting Igf2bp2 during mastitis. J. Agric. Food Chem. 71 (40), 14742–14757. 10.1021/acs.jafc.3c03350 37757458

[B52] Jiang S. Li H. Zhang L. Mu W. Zhang Y. Chen T. (2025). Generic Diagramming Platform (GDP): a comprehensive database of high-quality biomedical graphics. Nucleic Acids Res. 53 (D1), D1670–d1676. 10.1093/nar/gkae973 39470721 PMC11701665

[B53] Jin C. Jiang P. Zhang Z. Han Y. Wen X. Zheng L. (2024). Single-cell RNA sequencing reveals the pro-inflammatory roles of liver-resident Th1-like cells in primary biliary cholangitis. Nat. Commun. 15 (1), 8690. 10.1038/s41467-024-53104-9 39375367 PMC11458754

[B54] Johnston L. K. Bryce P. J. (2017). Understanding interleukin 33 and its roles in eosinophil development. Front. Med. (Lausanne) 4, 51. 10.3389/fmed.2017.00051 28512632 PMC5411415

[B55] Jorssen J. Van Hulst G. Mollers K. Pujol J. Petrellis G. Baptista A. P. (2024). Single-cell proteomics and transcriptomics capture eosinophil development and identify the role of IL-5 in their lineage transit amplification. Immunity 57 (7), 1549–1566.e8. 10.1016/j.immuni.2024.04.027 38776917

[B56] Kang S. Tanaka T. Narazaki M. Kishimoto T. (2019). Targeting interleukin-6 signaling in clinic. Immunity 50 (4), 1007–1023. 10.1016/j.immuni.2019.03.026 30995492

[B57] Karki R. Sharma B. R. Tuladhar S. Williams E. P. Zalduondo L. Samir P. (2021). Synergism of TNF-α and IFN-γ triggers inflammatory cell death, tissue damage, and mortality in SARS-CoV-2 infection and cytokine shock syndromes. Cell 184 (1), 149–168.e17. 10.1016/j.cell.2020.11.025 33278357 PMC7674074

[B58] Kim H. Banerjee N. Barnes R. C. Pfent C. M. Talcott S. T. Dashwood R. H. (2017). Mango polyphenolics reduce inflammation in intestinal colitis-involvement of the miR-126/PI3K/AKT/mTOR axis *in vitro* and *in vivo* . Mol. Carcinog. 56 (1), 197–207. 10.1002/mc.22484 27061150 PMC5053910

[B59] Kim T. S. Silva L. M. Theofilou V. I. Greenwell-Wild T. Li L. Williams D. W. (2023). Neutrophil extracellular traps and extracellular histones potentiate IL-17 inflammation in periodontitis. J. Exp. Med. 220 (9), e20221751. 10.1084/jem.20221751 37261457 PMC10236943

[B60] Koksal H. Vatansev H. Artac H. Kadoglou N. (2020). The clinical value of interleukins-8, -10, and -17 in idiopathic granulomatous mastitis. Clin. Rheumatol. 39 (5), 1671–1677. 10.1007/s10067-020-04925-8 31916110

[B61] Kong C. Zhang C. Wu Y. Zeng Z. Yu H. Zeng J. (2020). The expression and meaning of CD68, CD163, CD57, and IgG4 in granulomatous lobular mastitis. Gland. Surg. 9 (4), 936–949. 10.21037/gs-20-419 32953603 PMC7475371

[B62] Krausgruber T. Redl A. Barreca D. Doberer K. Romanovskaia D. Dobnikar L. (2023). Single-cell and spatial transcriptomics reveal aberrant lymphoid developmental programs driving granuloma formation. Immunity 56 (2), 289–306.e7. 10.1016/j.immuni.2023.01.014 36750099 PMC9942876

[B63] Lai J. L. Liu Y. H. Liu C. Qi M. P. Liu R. N. Zhu X. F. (2017). Indirubin inhibits LPS-induced inflammation *via* TLR4 abrogation mediated by the NF-kB and MAPK signaling pathways. Inflammation 40 (1), 1–12. 10.1007/s10753-016-0447-7 27718095

[B64] Langrish C. L. Chen Y. Blumenschein W. M. Mattson J. Basham B. Sedgwick J. D. (2005). IL-23 drives a pathogenic T cell population that induces autoimmune inflammation. J. Exp. Med. 201 (2), 233–240. 10.1084/jem.20041257 15657292 PMC2212798

[B65] Lee G. R. (2018). The balance of Th17 *versus* treg cells in autoimmunity. Int. J. Mol. Sci. 19 (3), 730. 10.3390/ijms19030730 29510522 PMC5877591

[B66] Li X. Q. Sun H. G. Wang X. H. Zhang H. J. Zhang X. S. Yu Y. (2022a). Activation of C3 and C5 may be involved in the inflammatory progression of PCM and GM. Inflammation 45 (2), 739–752. 10.1007/s10753-021-01580-2 34997873

[B67] Li C. Yao M. Li X. Shao S. Chen J. Li G. (2022b). Ultrasonic multimodality imaging features and the classification value of nonpuerperal mastitis. J. Clin. Ultrasound 50 (5), 675–684. 10.1002/jcu.23205 35475482

[B68] Li X. Xiao S. Filipczak N. Yalamarty S. S. K. Shang H. Zhang J. (2023). Role and therapeutic targeting strategies of neutrophil extracellular traps in inflammation. Int. J. Nanomedicine 18, 5265–5287. 10.2147/ijn.S418259 37746050 PMC10516212

[B69] Liew P. X. Kubes P. (2019). The neutrophil's role during health and disease. Physiol. Rev. 99 (2), 1223–1248. 10.1152/physrev.00012.2018 30758246

[B70] Liew F. Y. Girard J. P. Turnquist H. R. (2016). Interleukin-33 in health and disease. Nat. Rev. Immunol. 16 (11), 676–689. 10.1038/nri.2016.95 27640624

[B71] Liu Y. Zhang J. Zhou Y. H. Jiang Y. N. Zhang W. Tang X. J. (2015). IL-6/STAT3 signaling pathway is activated in plasma cell mastitis. Int. J. Clin. Exp. Pathol. 8 (10), 12541–12548. 26722442 PMC4680387

[B72] Liu L. Zhou F. Wang P. Yu L. Ma Z. Li Y. (2017). Periductal mastitis: an inflammatory disease related to bacterial infection and consequent immune responses? Mediat. Inflamm. 2017, 5309081. 10.1155/2017/5309081 28182101 PMC5274658

[B73] Liu Y. Zhang J. Zhou Y. H. Zhang H. M. Wang K. Ren Y. (2018). Activation of the IL-6/JAK2/STAT3 pathway induces plasma cell mastitis in mice. Cytokine 110, 150–158. 10.1016/j.cyto.2018.05.002 29751177

[B74] Liu Y. Sun Y. Zhou Y. Tang X. Wang K. Ren Y. (2020). Sinomenine hydrochloride inhibits the progression of plasma cell mastitis by regulating IL-6/JAK2/STAT3 pathway. Int. Immunopharmacol. 81, 106025. 10.1016/j.intimp.2019.106025 31810886

[B75] Liu J. Cheng Y. Zhang X. Chen Y. Zhu H. Chen K. (2023a). Glycosyltransferase Extl1 promotes CCR7-mediated dendritic cell migration to restrain infection and autoimmunity. Cell Rep. 42 (1), 111991. 10.1016/j.celrep.2023.111991 36656709

[B76] Liu C. Yu H. Chen G. Yang Q. Wang Z. Niu N. (2023b). An herbal drug combination identified by knowledge graph alleviates the clinical symptoms of plasma cell mastitis patients: a nonrandomized controlled trial. Elife 12, e84414. 10.7554/eLife.84414 36917037 PMC10063228

[B77] Long Q. Fan Y. Zhang J. Li H. Lv Q. (2021). Pruritic breast mass with palpable lymph nodes in a male patient: a case report. Gland. Surg. 10 (2), 826–831. 10.21037/gs-21-40 33708564 PMC7944058

[B78] Lu J. Gu B. Han X. Feng Y. (2023). Mammary epithelial cell-derived exosomal miR-155-inhibitor played a key role in the treatment of mastitis *via* down-regulation of TLRs/NF-κB signaling pathway to inhibit inflammatory response. Cell Mol. Biol. (Noisy-le-grand) 69 (15), 160–166. 10.14715/cmb/2023.69.15.28 38279456

[B79] Lukens J. R. Gross J. M. Kanneganti T. D. (2012). IL-1 family cytokines trigger sterile inflammatory disease. Front. Immunol. 3, 315. 10.3389/fimmu.2012.00315 23087690 PMC3466588

[B80] Ma F. Xiao Y. Qian L. Zhang S. (2025). Intervention study of Yanghe decoction on plasma cell mastitis based on mammary microecology and metabolomics investigation. J. Pharm. Biomed. Anal. 262, 116870. 10.1016/j.jpba.2025.116870 40209500

[B81] Martín-Fontecha A. Thomsen L. L. Brett S. Gerard C. Lipp M. Lanzavecchia A. (2004). Induced recruitment of NK cells to lymph nodes provides IFN-gamma for T(H)1 priming. Nat. Immunol. 5 (12), 1260–1265. 10.1038/ni1138 15531883

[B82] Mathias J. R. Perrin B. J. Liu T. X. Kanki J. Look A. T. Huttenlocher A. (2006). Resolution of inflammation by retrograde chemotaxis of neutrophils in transgenic zebrafish. J. Leukoc. Biol. 80 (6), 1281–1288. 10.1189/jlb.0506346 16963624

[B83] McIntire K. R. Sell S. Miller J. F. (1964). Pathogenesis of the post-neonatal thymectomy wasting syndrome. Nature 204, 151–155. 10.1038/204151a0 14222260

[B84] McKenzie A. N. Culpepper J. A. de Waal Malefyt R. Brière F. Punnonen J. Aversa G. (1993). Interleukin 13, a T-cell-derived cytokine that regulates human monocyte and B-cell function. Proc. Natl. Acad. Sci. U. S. A. 90 (8), 3735–3739. 10.1073/pnas.90.8.3735 8097324 PMC46376

[B85] Mihlan M. Wissmann S. Gavrilov A. Kaltenbach L. Britz M. Franke K. (2024). Neutrophil trapping and nexocytosis, mast cell-mediated processes for inflammatory signal relay. Cell 187 (19), 5316–5335.e28. 10.1016/j.cell.2024.07.014 39096902

[B86] Mitroi G. G. Pleșea E. L. Mitroi G. F. Mitroi M. R. Neagoe C. D. Ianoși S. L. (2024). Exploring the potential of IL-4 and IL-13 plasma levels as biomarkers in atopic dermatitis. Life (Basel) 14 (3), 352. 10.3390/life14030352 38541676 PMC10971867

[B87] Moro-García M. A. Mayo J. C. Sainz R. M. Alonso-Arias R. (2018). Influence of inflammation in the process of T lymphocyte differentiation: proliferative, metabolic, and oxidative changes. Front. Immunol. 9, 339. 10.3389/fimmu.2018.00339 29545794 PMC5839096

[B88] Mousset A. Lecorgne E. Bourget I. Lopez P. Jenovai K. Cherfils-Vicini J. (2023). Neutrophil extracellular traps formed during chemotherapy confer treatment resistance *via* TGF-β activation. Cancer Cell 41 (4), 757–775.e10. 10.1016/j.ccell.2023.03.008 37037615 PMC10228050

[B89] Nakae S. Komiyama Y. Nambu A. Sudo K. Iwase M. Homma I. (2002). Antigen-specific T cell sensitization is impaired in IL-17-deficient mice, causing suppression of allergic cellular and humoral responses. Immunity 17 (3), 375–387. 10.1016/s1074-7613(02)00391-6 12354389

[B90] Nakagome K. Nagata M. (2024). The possible roles of IL-4/IL-13 in the development of eosinophil-predominant severe asthma. Biomolecules 14 (5), 546. 10.3390/biom14050546 38785953 PMC11117569

[B91] Nakayama T. Hirahara K. Onodera A. Endo Y. Hosokawa H. Shinoda K. (2017). Th2 cells in health and disease. Annu. Rev. Immunol. 35, 53–84. 10.1146/annurev-immunol-051116-052350 27912316

[B92] Nan Q. Qinnan W. Chaoqun M. A. Dexuan C. Haidong C. Yaoyao L. U. (2022). Mechanism underlying efficacy of Shugan Sanjie decoction on plasma cell mastitis, based on network pharmacology and experimental verification. J. Tradit. Chin. Med. 42 (3), 400–407. 10.19852/j.cnki.jtcm.20220311.004 35610009 PMC9924697

[B93] Ni Q. T. Han G. H. Pan C. (2025). Single-cell transcriptomics reveals an abnormal immune microenvironment in plasma cell mastitis. Ann. Med. 57 (1), 14. 10.1080/07853890.2024.2446694 41421796 PMC11702992

[B94] Ortiz-Mendoza C. M. Sánchez N. A. A. Dircio A. C. (2018). Fine-needle aspiration cytology to identify a rare mimicker of breast cancer: plasma cell mastitis. Rev. Bras. Ginecol. Obstet. 40 (8), 491–493. 10.1055/s-0038-1666809 29986351 PMC10309336

[B95] Pal B. Chen Y. Vaillant F. Capaldo B. D. Joyce R. Song X. (2021). A single-cell RNA expression atlas of normal, preneoplastic and tumorigenic states in the human breast. Embo J. 40 (11), e107333. 10.15252/embj.2020107333 33950524 PMC8167363

[B96] Park J. Wysocki R. W. Amoozgar Z. Maiorino L. Fein M. R. Jorns J. (2016). Cancer cells induce metastasis-supporting neutrophil extracellular DNA traps. Sci. Transl. Med. 8 (361), 361ra138. 10.1126/scitranslmed.aag1711 27798263 PMC5550900

[B97] Pène J. Rousset F. Brière F. Chrétien I. Bonnefoy J. Y. Spits H. (1988). IgE production by normal human lymphocytes is induced by interleukin 4 and suppressed by interferons gamma and alpha and prostaglandin E2. Proc. Natl. Acad. Sci. U. S. A. 85 (18), 6880–6884. 10.1073/pnas.85.18.6880 2970644 PMC282082

[B98] Peters F. Schuth W. (1989). Hyperprolactinemia and nonpuerperal mastitis (duct ectasia). Jama 261 (11), 1618–1620. 10.1001/jama.1989.03420110094030 2918655

[B99] Phu T. A. Ng M. Vu N. K. Bouchareychas L. Raffai R. L. (2022). IL-4 polarized human macrophage exosomes control cardiometabolic inflammation and diabetes in obesity. Mol. Ther. 30 (6), 2274–2297. 10.1016/j.ymthe.2022.03.008 35292359 PMC9171286

[B100] Pilsczek F. H. Salina D. Poon K. K. H. Fahey C. Yipp B. G. Sibley C. D. (2010). A novel mechanism of rapid nuclear neutrophil extracellular trap formation in response to *Staphylococcus aureus* . J. Immunol. 185 (12), 7413–7425. 10.4049/jimmunol.1000675 21098229

[B101] Proto J. D. Doran A. C. Gusarova G. Yurdagul A. Sozen E. Subramanian M. (2018). Regulatory T cells promote macrophage efferocytosis during inflammation resolution. Immunity 49 (4), 666–677.e6. 10.1016/j.immuni.2018.07.015 30291029 PMC6192849

[B102] Punnonen J. de Vries J. E. (1994). IL-13 induces proliferation, Ig isotype switching, and Ig synthesis by immature human fetal B cells. J. Immunol. 152 (3), 1094–1102. 10.4049/jimmunol.152.3.1094 7507958

[B103] Rahal O. M. Wolfe A. R. Mandal P. K. Larson R. Tin S. Jimenez C. (2018). Blocking interleukin (IL)4- and IL13-mediated phosphorylation of STAT6 (Tyr641) decreases M2 polarization of macrophages and protects against macrophage-mediated radioresistance of inflammatory breast cancer. Int. J. Radiat. Oncol. Biol. Phys. 100 (4), 1034–1043. 10.1016/j.ijrobp.2017.11.043 29485045

[B104] Reina-Campos M. Scharping N. E. Goldrath A. W. (2021). CD8(+) T cell metabolism in infection and cancer. Nat. Rev. Immunol. 21 (11), 718–738. 10.1038/s41577-021-00537-8 33981085 PMC8806153

[B105] Ren Y. Xu J. Li X. Zhao B. Zhang J. Zhang J. (2021). Sequential occurrence of different subtypes of nonpuerperal mastitis in contralateral breasts: a report of two cases. Int. J. Clin. Exp. Pathol. 14 (6), 782–785. 34239681 PMC8255195

[B106] Risager R. Bentzon N. (2010). Smoking and increased risk of mastitis. Ugeskr. Laeger 172 (33), 2218–2221. 20727287

[B107] Ruterbusch M. Pruner K. B. Shehata L. Pepper M. (2020). *In vivo* CD4(+) T cell differentiation and function: revisiting the Th1/Th2 paradigm. Annu. Rev. Immunol. 38, 705–725. 10.1146/annurev-immunol-103019-085803 32340571

[B108] Saydam M. Yilmaz K. B. Sahin M. Yanik H. Akinci M. Yilmaz I. (2021). New findings on autoimmune etiology of idiopathic granulomatous mastitis: Serum IL-17, IL-22 and IL-23 levels of patients. J. Invest Surg. 34 (9), 993–997. 10.1080/08941939.2020.1725190 32046543

[B109] Shapouri-Moghaddam A. Mohammadian S. Vazini H. Taghadosi M. Esmaeili S. A. Mardani F. (2018). Macrophage plasticity, polarization, and function in health and disease. J. Cell Physiol. 233 (9), 6425–6440. 10.1002/jcp.26429 29319160 PMC13482560

[B110] Sharabi A. Tsokos G. C. (2020). T cell metabolism: new insights in systemic lupus erythematosus pathogenesis and therapy. Nat. Rev. Rheumatol. 16 (2), 100–112. 10.1038/s41584-019-0356-x 31949287

[B111] Sharif S. Arreaza G. A. Zucker P. Mi Q. S. Sondhi J. Naidenko O. V. (2001). Activation of natural killer T cells by alpha-galactosylceramide treatment prevents the onset and recurrence of autoimmune type 1 diabetes. Nat. Med. 7 (9), 1057–1062. 10.1038/nm0901-1057 11533711

[B112] Shekhar S. Yang X. (2012). The darker side of follicular helper T cells: from autoimmunity to immunodeficiency. Cell Mol. Immunol. 9 (5), 380–385. 10.1038/cmi.2012.26 22885524 PMC4002332

[B113] Sica A. Mantovani A. (2012). Macrophage plasticity and polarization: *in vivo* veritas. J. Clin. Invest 122 (3), 787–795. 10.1172/jci59643 22378047 PMC3287223

[B114] Smithgall M. D. Comeau M. R. Yoon B. R. P. Kaufman D. Armitage R. Smith D. E. (2008). IL-33 amplifies both Th1- and Th2-type responses through its activity on human basophils, allergen-reactive Th2 cells, iNKT and NK cells. Int. Immunol. 20 (8), 1019–1030. 10.1093/intimm/dxn060 18550585

[B115] Sun X. Liu L. Wang J. Luo X. Wang M. Wang C. (2024a). Targeting STING in dendritic cells alleviates psoriatic inflammation by suppressing IL-17A production. Cell Mol. Immunol. 21 (7), 738–751. 10.1038/s41423-024-01160-y 38806624 PMC11214627

[B116] Sun X. Hou J. Ni T. Xu Z. Yan W. Kong L. (2024b). MCC950 attenuates plasma cell mastitis in an MDSC-dependent manner. Int. Immunopharmacol. 131, 111803. 10.1016/j.intimp.2024.111803 38460298

[B117] Thacker G. Henry S. Nandi A. Debnath R. Singh S. Nayak A. (2023). Immature natural killer cells promote progression of triple-negative breast cancer. Sci. Transl. Med. 15 (686), eabl4414. 10.1126/scitranslmed.abl4414 36888695 PMC10875969

[B118] Tsuda Y. Takahashi H. Kobayashi M. Hanafusa T. Herndon D. N. Suzuki F. (2004). Three different neutrophil subsets exhibited in mice with different susceptibilities to infection by methicillin-resistant *Staphylococcus aureus* . Immunity 21 (2), 215–226. 10.1016/j.immuni.2004.07.006 15308102

[B119] Tyagi A. Sharma S. Wu K. Wu S. Y. Xing F. Liu Y. (2021). Nicotine promotes breast cancer metastasis by stimulating N2 neutrophils and generating pre-metastatic niche in lung. Nat. Commun. 12 (1), 474. 10.1038/s41467-020-20733-9 33473115 PMC7817836

[B120] Ucaryilmaz H. Koksal H. Emsen A. Kadoglou N. Dixon J. M. Artac H. (2022). The role of regulatory T and B cells in the etiopathogenesis of idiopathic granulomatous mastitis. Immunol. Invest 51 (2), 357–367. 10.1080/08820139.2020.1832114 33034215

[B121] Valent P. (2009). Interleukin-33: a regulator of basophils. Blood 113 (7), 1396–1397. 10.1182/blood-2008-11-189811 19221042

[B122] Van den Bossche J. Baardman J. Otto N. A. van der Velden S. Neele A. E. van den Berg S. M. (2016). Mitochondrial dysfunction prevents repolarization of inflammatory macrophages. Cell Rep. 17 (3), 684–696. 10.1016/j.celrep.2016.09.008 27732846

[B123] Vervliet T. Parys J. B. Bultynck G. (2016). Bcl-2 proteins and calcium signaling: complexity beneath the surface. Oncogene 35 (39), 5079–5092. 10.1038/onc.2016.31 26973249

[B124] Vitenberga-Verza Z. Pilmane M. Šerstņova K. Melderis I. Gontar Ł. Kochański M. (2022). Identification of inflammatory and regulatory cytokines IL-1α-IL-4-IL-6-IL-12-IL-13-IL-17A-TNF-α-and IFN-γ-Producing cells in the milk of dairy cows with subclinical and clinical mastitis. Pathogens 11 (3), 372. 10.3390/pathogens11030372 35335696 PMC8954094

[B125] Wang J. Hossain M. Thanabalasuriar A. Gunzer M. Meininger C. Kubes P. (2017). Visualizing the function and fate of neutrophils in sterile injury and repair. Science 358 (6359), 111–116. 10.1126/science.aam9690 28983053

[B126] Wang Z. Wang N. Liu X. Wang Q. Xu B. Liu P. (2018). Broadleaf Mahonia attenuates granulomatous lobular mastitis-associated inflammation by inhibiting CCL-5 expression in macrophages. Int. J. Mol. Med. 41 (1), 340–352. 10.3892/ijmm.2017.3246 29138800 PMC5746325

[B127] Wang X. Han Y. Liu J. Zhang Y. Cheng K. Guo J. (2019). Exosomes play an important role in the progression of plasma cell mastitis *via* the PI3K-Akt-mTOR signaling pathway. Mediat. Inflamm. 2019, 4312016. 10.1155/2019/4312016 31281227 PMC6590603

[B128] Wang X. Qu Y. Xu Q. Jiang Z. Wang H. Lin B. (2024). NQO1 triggers neutrophil recruitment and NET formation to drive lung metastasis of invasive breast cancer. Cancer Res. 84 (21), 3538–3555. 10.1158/0008-5472.Can-24-0291 39073320

[B129] Worbs T. Hammerschmidt S. I. Förster R. (2017). Dendritic cell migration in health and disease. Nat. Rev. Immunol. 17 (1), 30–48. 10.1038/nri.2016.116 27890914

[B130] Wu Z. Yang Q. Ma H. (2022). Study the mechanism of Gualou Niubang Decoction in treating plasma cell mastitis based on network pharmacology and molecular docking. Biomed. Res. Int. 2022, 5780936. 10.1155/2022/5780936 35757473 PMC9217541

[B131] Xiao Y. Cong M. Li J. He D. Wu Q. Tian P. (2021). Cathepsin C promotes breast cancer lung metastasis by modulating neutrophil infiltration and neutrophil extracellular trap formation. Cancer Cell 39 (3), 423–437.e7. 10.1016/j.ccell.2020.12.012 33450198

[B132] Xie Y. Zhou T. Li X. Zhao K. Bai W. Hou X. (2024). Targeting ESE3/EHF with Nifurtimox inhibits CXCR2(+) neutrophil infiltration and overcomes pancreatic cancer resistance to chemotherapy and immunotherapy. Gastroenterology 167 (2), 281–297. 10.1053/j.gastro.2024.02.046 38492894

[B133] Xie L. Feng J. Gao Q. Qu W. Shao S. Sun J. (2025). The autoimmune profiles in the etiopathogenesis of granulomatous lobular mastitis. Immunobiology 230 (2), 152878. 10.1016/j.imbio.2025.152878 39922144

[B134] Xu Y. J. Da B. Zhao F. Gao M. Xue L. Zheng H. (2023). Corrective surgery for nipple depression in patients with plasmacytic mastitis - a single-center experience. Front. Med. 10, 1156628. 10.3389/fmed.2023.1156628 37089608 PMC10118005

[B135] Yang Y. Huang Y. Li P. Hu J. Jiang B. Zhou X. (2018). Differential diagnosis for breast ductal carcinoma *in situ* and plasma cell mastitis by magnetic resonance imaging. Zhong Nan Da Xue Xue Bao Yi Xue Ban. 43 (10), 1123–1130. 10.11817/j.issn.1672-7347.2018.10.013 30523234

[B136] Yang J. Yang X. Yang J. Li M. (2019). Baicalin ameliorates lupus autoimmunity by inhibiting differentiation of Tfh cells and inducing expansion of Tfr cells. Cell Death Dis. 10 (2), 140. 10.1038/s41419-019-1315-9 30760702 PMC6374440

[B137] Yigitbasi M. R. Guntas G. Atak T. Sonmez C. Yalman H. Uzun H. (2017). The role of Interleukin-33 as an inflammatory marker in differential diagnosis of idiopathic granulomatous mastitis and breast cancer. J. Invest Surg. 30 (4), 272–276. 10.1080/08941939.2016.1240270 27780363

[B138] York A. G. Skadow M. H. Oh J. Qu R. Zhou Q. D. Hsieh W. Y. (2024). IL-10 constrains sphingolipid metabolism to limit inflammation. Nature 627 (8004), 628–635. 10.1038/s41586-024-07098-5 38383790 PMC10954550

[B139] Yu C. Zhang C. Huai Y. Liu D. Zhang M. Wang H. (2024). The inhibition effect of caffeic acid on NOX/ROS-dependent macrophages M1-like polarization contributes to relieve the LPS-induced mice mastitis. Cytokine 174, 156471. 10.1016/j.cyto.2023.156471 38103301

[B140] Yuan Q. Q. Xiao S. Y. Farouk O. Du Y. T. Sheybani F. Tan Q. T. (2022). Management of granulomatous lobular mastitis: an international multidisciplinary consensus (2021 edition). Mil. Med. Res. 9 (1), 20. 10.1186/s40779-022-00380-5 35473758 PMC9040252

[B141] Yunna C. Mengru H. Lei W. Weidong C. (2020). Macrophage M1/M2 polarization. Eur. J. Pharmacol. 877, 173090. 10.1016/j.ejphar.2020.173090 32234529

[B142] Zhang Y. Zhou Y. Mao F. Guan J. Sun Q. (2018). Clinical characteristics, classification and surgical treatment of periductal mastitis. J. Thorac. Dis. 10 (4), 2420–2427. 10.21037/jtd.2018.04.22 29850148 PMC5949503

[B143] Zhang R. Qi C. F. Hu Y. Shan Y. Hsieh Y. P. Xu F. (2019). T follicular helper cells restricted by IRF8 contribute to T cell-mediated inflammation. J. Autoimmun. 96, 113–122. 10.1016/j.jaut.2018.09.001 30241692 PMC6310655

[B144] Zhang H. J. Ding P. P. Zhang X. S. Wang X. C. Sun D. W. Bu Q. A. (2022). MAC mediates mammary duct epithelial cell injury in plasma cell mastitis and granulomatous mastitis. Int. Immunopharmacol. 113 (Pt A), 109303. 10.1016/j.intimp.2022.109303 36252469

[B145] Zhang H. Wang Y. Qu M. Li W. Wu D. Cata J. P. (2023). Neutrophil, neutrophil extracellular traps and endothelial cell dysfunction in sepsis. Clin. Transl. Med. 13 (1), e1170. 10.1002/ctm2.1170 36629024 PMC9832433

[B146] Zhang H. Wu D. Wang Y. Shi Y. Shao Y. Zeng F. (2024a). Ferritin-mediated neutrophil extracellular traps formation and cytokine storm *via* macrophage scavenger receptor in sepsis-associated lung injury. Cell Commun. Signal 22 (1), 97. 10.1186/s12964-023-01440-6 38308264 PMC10837893

[B147] Zhang B. Liu J. Mo Y. Zhang K. Huang B. Shang D. (2024b). CD8(+) T cell exhaustion and its regulatory mechanisms in the tumor microenvironment: key to the success of immunotherapy. Front. Immunol. 15, 1476904. 10.3389/fimmu.2024.1476904 39372416 PMC11452849

[B148] Zhao X. Di Q. Liu H. Quan J. Ling J. Zhao Z. (2022). MEF2C promotes M1 macrophage polarization and Th1 responses. Cell Mol. Immunol. 19 (4), 540–553. 10.1038/s41423-022-00841-w 35194174 PMC8975968

[B149] Zhao J. Ji H. Wang X. Wang Y. Xia Z. (2023). Association of Nonpuerperal Mastitis with cytokines related to helper T cells TH1/TH2 and TH17/Treg. Altern. Ther. Health Med. 29 (8), 150–155. 37535921

[B150] Zheng Y. Wang L. Han X. Shen L. Ling C. Qian Z. (2022a). Combining contrast-enhanced ultrasound and blood cell analysis to improve diagnostic accuracy of plasma cell mastitis. Exp. Biol. Med. (Maywood) 247 (2), 97–105. 10.1177/15353702211049361 34632855 PMC8777476

[B151] Zheng B. Song J. Lu M. Chen C. Sun S. (2022b). Current research describing the role of CD4(+) T lymphocyte subsets in the pathogenesis of granulomatous lobular mastitis. J. Invest Surg. 35 (10), 1790–1795. 10.1080/08941939.2022.2090035 36075587

[B152] Zhou Y. Feng B. J. Yue W. W. Liu Y. Xu Z. F. Xing W. (2022). Differentiating non-lactating mastitis and malignant breast tumors by deep-learning based AI automatic classification system: a preliminary study. Front. Oncol. 12, 997306. 10.3389/fonc.2022.997306 36185190 PMC9521279

[B153] Zhou Y. Xu Z. F. Xing W. Liu Y. Xu Z. Tan G. L. (2023). Comparative study of ultrasound-guided microwave ablation and traditional surgery in the treatment of plasma cell mastitis: a multicenter study. Quant. Imaging Med. Surg. 13 (3), 1838–1848. 10.21037/qims-21-1132 36915313 PMC10006106

[B154] Zhou F. Li H. Wang F. Liu L. Yu L. Xiang Y. (2024a). Efficacy and safety of rifampicin-based triple therapy for non-puerperal mastitis: a single-arm, open-label, prospective clinical trial. Int. J. Infect. Dis. 140, 25–30. 10.1016/j.ijid.2023.12.008 38142735

[B155] Zhou Y. Gong J. Deng X. Shen L. Liu L. (2024b). Novel insights: crosstalk with non-puerperal mastitis and immunity. Front. Immunol. 15, 1431681. 10.3389/fimmu.2024.1431681 39148739 PMC11324573

[B156] Zhou F. Liu L. Wang F. Yu L. Xiang Y. Zheng C. (2024c). Periductal mastitis, a disease with distinct clinicopathological features from granulomatous lobular mastitis. J. Inflamm. Res. 17, 3815–3823. 10.2147/jir.S464585 38895142 PMC11185250

[B157] Zhou Y. Deng X. Ruan H. Xue X. Hu Z. Gong J. (2025). Single-cell RNA sequencing reveals the immune landscape of granulomatous mastitis. Inflammation 48, 4046–4061. 10.1007/s10753-025-02310-8 40338490 PMC12722302

[B158] Zhu J. (2015). T helper 2 (Th2) cell differentiation, type 2 innate lymphoid cell (ILC2) development and regulation of interleukin-4 (IL-4) and IL-13 production. Cytokine 75 (1), 14–24. 10.1016/j.cyto.2015.05.010 26044597 PMC4532589

[B159] Zhu Y. C. Zhang Y. Deng S. H. Jiang Q. Shi X. R. Feng L. L. (2019). Evaluation of plasma cell mastitis with superb microvascular imaging. Clin. Hemorheol. Microcirc. 72 (2), 129–138. 10.3233/ch-180468 30636730

[B160] Zhu S. Yu Y. Qu M. Qiu Z. Zhang H. Miao C. (2023). Neutrophil extracellular traps contribute to immunothrombosis formation *via* the STING pathway in sepsis-associated lung injury. Cell Death Discov. 9 (1), 315. 10.1038/s41420-023-01614-8 37626060 PMC10457383

[B161] Zindel J. Kubes P. (2020). DAMPs, PAMPs, and LAMPs in immunity and sterile inflammation. Annu. Rev. Pathol. 15, 493–518. 10.1146/annurev-pathmechdis-012419-032847 31675482

[B162] Zitti B. Bryceson Y. T. (2018). Natural killer cells in inflammation and autoimmunity. Cytokine Growth Factor Rev. 42, 37–46. 10.1016/j.cytogfr.2018.08.001 30122459

[B163] Zuo X. M. Wang T. S. Shi X. G. Gao X. Gao S. Sun P. (2021). Pyroptosis: the pathological process that dominates granulomatous lobular mastitis. J. Physiol. Pharmacol. 72 (3). 10.26402/jpp.2021.3.15 34873070

